# High-plex spatial transcriptomic profiling reveals distinct immune components and the HLA class I/DNMT3A/CD8 modulatory axis in mismatch repair-deficient endometrial cancer

**DOI:** 10.1007/s13402-023-00885-8

**Published:** 2023-10-17

**Authors:** Jingjing Guo, Baijie Tang, Jing Fu, Xuan Zhu, Wenlong Xie, Nan Wang, Zhiyong Ding, Zhentao Song, Yue Yang, Gang Xu, Xue Xiao

**Affiliations:** 1 Department of Pathology, Sichuan Provincial People’s Hospital, School of Medicine, University of Electronic Science and Technology of China, Chengdu, China; 2 School of Medical and Life Sciences, Chengdu University of TCM, Chengdu, China; 3 Mills Institute for Personalized Cancer Care, Jinan, China

**Keywords:** Mismatch repair deficiency (MMRd), Immunotherapy, Endometrial cancer, Digital spatial profiling, HLA class I, DNMT3A

## Abstract

**Purpose:**

Tumors bearing mismatch repair deficiency (MMRd) are characterized by a high load of neoantigens and are believed to trigger immunogenic reactions upon immune checkpoint blockade treatment such as anti-PD-1/PD-L1 therapy. However, the mechanisms are still ill-defined, as multiple cancers with MMRd exhibit variable responses to immune checkpoint inhibitors (ICIs). In endometrial cancer (EC), a distinct tumor microenvironment (TME) exists that may correspond to treatment-related efficacies. We aimed to characterize EC patients with aberrant MMR pathways to identify molecular subtypes predisposed to respond to ICI therapies.

**Methods:**

We applied digital spatial profiling, a high-plex spatial transcriptomic approach covering over 1,800 genes, to obtain a highly resolved TME landscape in 45 MMRd-EC patients. We cross-validated multiple biomarkers identified using immunohistochemistry and multiplexed immunofluorescence using in-study and independent cohorts totaling 123 MMRd-EC patients and validated our findings using external TCGA data from microsatellite instability endometrial cancer (MSI-EC) patients.

**Results:**

High-plex spatial profiling identified a 14-gene signature in the MMRd tumor-enriched regions stratifying tumors into “hot”, “intermediate” and “cold” groups according to their distinct immune profiles, a finding highly consistent with the corresponding CD8 + T-cell infiltration status. Our validation studies further corroborated an existing coregulatory network involving HLA class I and DNMT3A potentially bridged through dynamic crosstalk incorporating CCL5.

**Conclusion:**

Our study confirmed the heterogeneous TME status within MMRd-ECs and showed that these ECs can be stratified based on potential biomarkers such as HLA class I, DNMT3A and CD8 in pathological settings for improved ICI therapeutic efficacy in this subset of patients.

**Supplementary Information:**

The online version contains supplementary material available at 10.1007/s13402-023-00885-8.

## Introduction

Endometrial cancer (EC), as one of the leading gynecological malignancies, accounts for over 417,000 new-onset cases annually [1]. While most patients show excellent prognosis, there are several known factors for recurrent disease, such as histological type and grade. In-depth multiomics profiling of clinical specimens has revealed distinct molecular subtypes, namely, four predominant groups defined by DNA polymerase epsilon mutation (POLEm), mismatch repair protein deficiency (MMRd)/microsatellite instability (MSI), copy-number low and copy-number high [2–4]. Follow-up studies have revealed the prognostic value of molecularly stratified subgroups with high-copy-number patients showing dismal progression-free survival and cancer-specific survival, whereas the POLEm group exhibits optimal outcomes [5].

Although cumulative evidence highlights the importance of molecular classification in EC, less is known about its MMRd/MSI status with a more miscellaneous nature related to the tumor microenvironment (TME). The DNA mismatch repair (MMR) system plays an important role in maintaining genetic fidelity, and deficiency in this pathway increases the risk of multiple cancers [6, 7]. As such, tumors with MMRd are characterized by widespread MSI and high mutational burden, making them more immunogenetic and likely to respond to anti-PD-1/PD-L1 immunotherapy [8, 9]. However, despite a high load of neoantigens, patients with MMRd exhibit variable responses to immune checkpoint inhibitors (ICIs), and over half are resistant to this treatment [10, 11]. Focused studies on ECs have reported similar findings [12, 13]. Mechanism-based studies have uncovered the bona fide relationship between the degree of MSI and its impact on anti–PD-1 immunotherapy [14]. However, in MMRd cancers treated with pembrolizumab, the number of tumor nonsynonymous mutations is not significantly different between responders and nonresponders [9]. These observations indicate that in addition to hypermutation-mediated neoantigens, other factors in MMRd tumors may play roles in sensitivity to immune checkpoint inhibitors (ICIs).

More importantly, the dynamic regulation of the TME in EC has not been well established. Notably, an ample number of studies have already demonstrated the importance of preexisting CD8 + T cells in the TME for effective ICI intervention, and a lack of T-cell infiltration can make MMRd tumors insensitive to this treatment [15–17]. Since a subset of MMRd tumors displays a low density of tumor-infiltrating lymphocytes (TILs) [18, 19], these highly mutated tumors may harbor dysregulated pathways within the TME causing T-cell retention in peritumor regions. Recently, Vasaikar reported a negative correlation between glycolytic activity and infiltration of CD8 + T cells in MSI-H colon cancer, while Lu’s team demonstrated that deficiency of the cGAS-STING pathway in MMRd tumors dramatically diminishes CD8 + T-cell infiltration, suggesting probable underlying mechanisms to facilitate tumorigenesis with high mutational burden [17, 20]. Nevertheless, evidence is still sparse in MMRd-EC to address crucial mechanisms associated with T-cell infiltration in MMRd tumors, which may aid in the identification of predictive biomarkers for ICI as well as in the design of new strategies to overcome therapeutic resistance in this subgroup of ECs.

Herein, we applied the digital spatial profiler (DSP), a high-plex spatial biology discovery tool, to obtain a high-resolution TME transcription profile of MMRd-EC [21]. Through a comprehensive analysis of over 1,800 key tumor immune-related genes in 45 MMRd-EC patients, we identified a 14-gene signature in the tumor-specific region classifying tumors into “hot”, “intermediate” and “cold” groups according to their distinct immune activity and CD8 + T-cell infiltration. Additional validation using independent clinical cohorts confirmed a tight potential connection between HLA class I genes and DNMT3A that may be predictive of a response to anti-PD-1/PD-L1 therapy. Our findings elucidated an existing interpatient heterogeneity of MMRd-EC and discovered the key components via a dynamic association between HLA class I/DNMT3A expression and CD8 + T-cell infiltration status. These molecules may carry biomarker potential for stratification of MMRd-EC patients and identification of those likely to benefit from ICI treatment.

## Materials and methods

### Patient sample acquisition and pathological evaluation

A total of 653 patients receiving surgical resection for EC between 2013 and 2021 were preselected from the pathology archive of Sichuan Provincial People’s Hospital with ethical approval from an internal committee. Hematoxylin and eosin (H&E) staining was performed on formalin-fixed, paraffin-embedded (FFPE) sections, and pathological diagnosis was reviewed by two senior pathologists. Clinicopathological information was obtained from electronic medical records. Event-free survival (EFS) was defined as the time from diagnosis to the time of the first event (disease progression/relapse or disease-associated death).

### Immunohistochemistry, pathological assessment criteria and POLE sequencing

Immunohistochemistry (IHC) was conducted as described previously [19]. Staining of mismatch repair proteins (MLH1, MSH2, MSH6 and PMS2) was performed on all 653 samples, while IHC for PD-L1, HLA class I, and DNMT3A was performed on 123 MMRd/MSI samples only. CD8 staining was performed on 123 MMRd/MSI samples and 123 mismatch repair proficient (MMRp) samples (tumor grade matched with MMRd samples). MMRd/MSI was defined as complete loss of nuclear staining of any MMR protein in tumor cells with the presence of positive internal controls. Tumors with expression of all four MMR proteins were defined as MMRp. The density of CD8 + TILs was evaluated as the number of CD8 + lymphocytes located within the tumor epithelium. For each sample, the average count was determined from five randomly selected high-power fields. PD-L1 expression (clone SP142) in tumor cells (TCs) was scored based on the proportion of tumor area occupied by membranous stained TCs of any intensity. Positive TC expression of PD-L1 was defined as a TC score ≥ 1% [22, 23]. To assess the expression of HLA class I, an anti-HLA class I ABC antibody targeting HLA-A, B and C heavy chains was used. HLA class I positivity ( +) was defined as > 90% of TCs showing membranous and/or cytoplasmic expression; subclonal loss ( ±) was defined as 10–90% of TCs showing expression; and negative expression (-) was defined as < 10% of TCs showing expression of HLA class I [24]. Representative staining patterns of HLA class I are shown in Supplementary Fig. 1. DNMT3A expression in each case was evaluated using the weighted histoscore method, which is based on staining intensity and percentages of stained tumor cells [25]. Examples of weak, intermediate, and strong staining intensity are shown in Supplementary Fig. 2. The histoscore for each case was calculated as follows: 1 × percentage of cells with weak staining + 2 × percentage of cells with intermediate staining + 3 × percentage of cells with strong staining. The maximum score is 300. Samples with a histoscore of 0 were defined as negative expression (-); samples with a histoscore less than 200 were defined as weak expression ( +); samples with a histoscore equal to 200 were defined as moderate expression (+ +); and samples with a histoscore above 200 were defined as strong expression (+ + +). POLE sequencing was carried out on all MMRd patients using a HiSeq 2500 sequencer at an average depth of > 1,000 × .

### Selection of MMRd samples

**Fig. 1:**
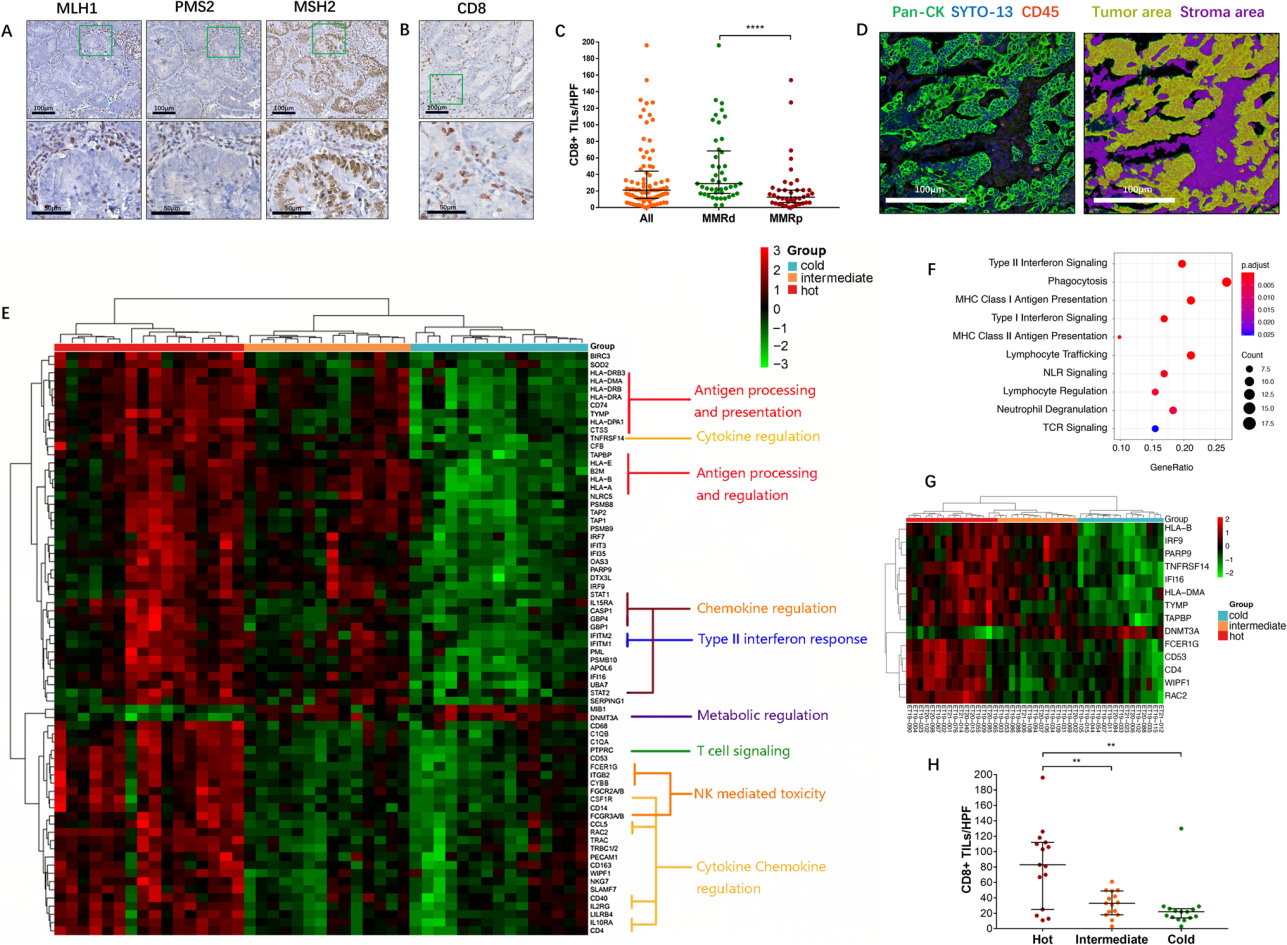
DSP spatial transcriptomic profiling of MMRd ECs. (**A**) A representative MMRd tumor with loss of MLH1 and PMS2 expression. The bottom row shows zoomed regions corresponding to the green boxes in the upper row. Magnification: 50 times (top) and 200 times (bottom). (**B**) A representative case with high CD8 + T-cell infiltration. The lower image shows higher magnification of the regions in the green box above. Magnification: 5 times (top) and 200 times (bottom). (**C**) Comparison of the density of CD8 + TILs between MMRd and MMRp tumors. The Mann‒Whitney test was used, with *p* < 0.05 indicating statistical significance. ****, *P* < 0.0001. Error bars indicate 1^st^ and 3^rd^ quartiles. (**D**) Representative images of ROIs indicating the tumor area and stromal area. The right image shows the segments of these two regions defined by DSP automatically. (**E**) Heatmap showing stratification of MMRd tumors into “hot”, “intermediate” and “cold” immune subgroups by the WGCNA-derived 70-gene signature. All transcript counts are scaled. (**F**) GO enrichment analysis showing the top 10 pathways of the 70 genes. (**G**) Heatmap showing the classification of MMRd tumors into the three immune subgroups by the 14 core genes. All transcript counts are shown in scaled format. (**H**) Comparison of the density of CD8 + TILs from hot, intermediate and cold tumors stratified by the 14-gene signature. The Kruskal‒Wallis test with Dunn's multiple comparisons test was used, with *p* < 0.05 indicating statistical significance. **, *P* < 0.01. Error bars indicate 1^st^ and 3^rd^ quartiles. Abbreviations: MMRd, MMR deficiency; MMRp, MMR proficiency; HPF, high-power field

To select EC patients for spatial profiling, 653 EC samples were screened with immunohistochemistry (IHC), and 141 (21.6%) MMRd tumors were identified according to their MMR protein expression status. Patients with neoadjuvant chemotherapy prior to surgery or with POLE mutation were excluded. Finally, 123 MMRd cases with sufficient tumor samples were selected for further study. The clinicopathological features of the 123 MMRd ECs are summarized in Supplementary Table 2. An example of an MMRd tumor is shown in Fig. 1A. Out of the 123 MMRd patients, 45 diagnosed between 2019 and 2021 (cohort 1) were used for digital spatial profiling, fluorescent multiplex immunohistochemistry (mIHC) and IHC characterization. For the remaining 78 EC patients diagnosed between 2013 and 2018 (cohort 2), only IHC experiments were conducted.

### Spatial transcriptome profiling using digital spatial profiling based on TMA

For digital spatial profiling (DSP), two 5 μm TMA (2 mm in diameter/core) sections representative of MMRd patients in cohort 1 (45 patients diagnosed between 2019 and 2021) were deparaffinized and rehydrated, followed by antigen retrieval and proteinase digestion following the DSP procedure. For each FFPE block, one 2 mm core from the tumor center was selected. Briefly, slides were incubated with the Cancer Transcriptome Atlas (CTA) probe set covering over 1,800 genes (NanoString) and stained with fluorescent morphology markers targeting PanCK (epithelial and tumoral regions), CD45 (immune cells), and SYTO-13 (nuclear). For each tumor, regions of interest (ROIs) representing PanCK + (tumor) and PanCK-/CD45 + (immune-stroma) areas were selected. Oligos (barcodes) targeting individual transcripts were photocleaved and collected into microwell plates for library preparation and sequencing.

### Fluorescent multiplex immunohistochemistry (mIHC) and image analysis

Multiplex IHC for Pan-CK, CD68, CD163, CD86, CD4 and Foxp3 was performed on TMAs (Supplementary Table 1). Slides were deparaffinized and rehydrated following heat-induced antigen retrieval, blocked with 3% H_2_O_2_ and 3% bovine serum albumin (BSA) and then costained with antibody panels (Pan-CK, CD68, CD163, and CD86; Pan-CK, CD4 and Foxp3). A TSA kit (Recordbio Biological Technology) was used for signal detection. Slides were scanned with a scanner (Pannoramic MIDI: 3Dhistech), and images were analyzed via HALO Highplex FL v4.1.3 (Indica Labs).

### Hub gene identification using WGCNA and functional enrichment

To leverage the analytical bias potentially associated with cutoff-based gene filtering, weighted gene coexpression network analysis (WGCNA) was applied. Tumor ROIs from MMRd patients in cohort 1 were selected for analysis. In general, 1,539 QC-filtered genes from DSP-CTA profiling were used as input. An R^2^ of 0.85 was used to determine the optimal soft threshold and mean connectivity. Transcriptional modules were then identified using dynamic cut with the minimal module containing 30 genes. All regulatory modules containing individual gene sets were then applied to find possible correlations with levels of CD8 + T-cell infiltration predefined as hot and cold according to the density of CD8 + TILs (cut-off point: 21 CD8 + lymphocytes/HPF, Supplementary Table 3). Co-clustered samples in the middle region defined by the 70-gene signature were then defined as intermediate. Most explainable modules were selected and filtered by their gene significance and module membership. For the resulting gene functional enrichment, WGCNA-derived module genes were used, and annotated pathway information derived from the Reactome database was applied (Supplementary Table 4).

### Core signature extraction using two-step lasso regression

The WGCNA-derived signature for stratifying MMRd ECs into hot, intermediate and cold immune subgroups was narrowed down using the lasso regression-based feature selection method (R package glmnet). In the first step, all MMRd tumor ROIs were assigned to either cold or “noncold” groups, and leave-one-out cross-validation was used to obtain the best lambda for gene feature determination. In the second step, noncold ROI regions containing hot and intermediate ROIs were used for lasso regression using the same strategy, resulting in a final gene signature for classifying patients with different levels of CD8 + TILs. The gene signature for stratifying MMRd-ECs was orthogonally validated using a deconvolution-based immune cell typing algorithm (R package SpatialDecon) [26].

### Cross-validation of spatial transcriptomic-defined immune subgroups using TCGA data

Transcriptome profiling, microsatellite stability data and clinical data of endometrial cancers from the TCGA cohort (TCGA-UCEC) were downloaded (TCGAbiolinks R package). Transcription data processing followed the STAR method, and upper quartile normalized data were used for downstream analysis. The CIBERSORT method was applied to the preprocessed transcriptomic data to deconvolute cell types and their fractions correspondingly. The 14-gene signature derived from WGCNA and subsequent two-step lasso regression were projected onto the bulk transcriptomic dataset to obtain a subset expression matrix and used to allocate TCGA MSI-H patients into the hot, intermediate and cold groups using the parameters from the two-step lasso regression model.

### Statistical analysis

Statistical analysis was performed with R and GraphPad Prism 7. Fisher’s exact test and the chi-square test were used for cross-tables. The Mann–Whitney U test and Kruskal–Wallis test were used to analyze groups of unpaired variables. Pearson correlation analysis was used to measure the linear correlation between two groups of variables. Survival curves were computed using the Kaplan–Meier method, and statistical significance was determined using the log-rank test. A probability value of *p* < 0.05 was considered statistically significant, and two-tailed p values were reported for two-group comparisons.

## Results

### Spatial transcriptomic-based MMRd patient stratification using DSP

**Fig. 2:**
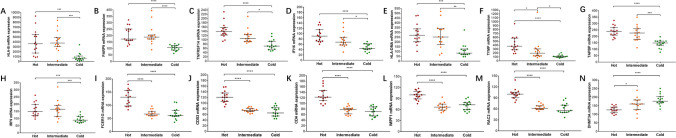
Analysis of the expression of the 14 genes according to the three immune subtypes and the density of CD8 + TILs. Comparison of the expression of HLA-B (**A**), PAPR9 (**B**), TNFRSF14 (**C**), IFI16 (**D**), HLA-DMA (**E**), TYMP (F), TAPBP (**G**), IRF9 (H), FCER1G (**I**), CD53 (**J**), CD4 (**K**), WIPF1 (**L**), RAC2 (**M**) and DNMT3A (**N**) in “hot”, “intermediate” and “cold” tumors. The Kruskal‒Wallis test with Dunn's multiple comparisons test was used with *p* < 0.05 indicating statistical significance. *, *P* < 0.05, **, *P* < 0.01; ***, *P* < 0.001; ****, *P* < 0.0001

We first characterized the four MMR proteins (MLH1, PMS2, MSH2 and MSH6) together with CD8 + T-cell infiltration levels in ECs using IHC (Fig. 1A). We observed a higher level of CD8 + T-cell infiltration in MMRd tumors than in MMRp tumors; however, a small proportion of MMRd tumors displayed a low density of CD8 + TILs, indicating the heterogeneous nature of MMRd EC (Fig. 1B/C). Therefore, we conducted deep spatial profiling of 45 preselected MMRd patients using DSP to identify altered transcriptomic programs associated with the level of CD8 + T-cell infiltration. By quantitatively analyzing over 1,800 key immuno-oncology-related genes in a spatially directed manner, a deep tumor-immune profile was established within the tumor (PanCK +) and stromal (CD45 +) regions (Fig. 1D). Subsequently, we applied the WGCNA method to find the core transcriptional programs associated with tumor regions to resolve the intratumoral heterogeneity in MMRd EC. (Supplementary Fig. 3). We identified 5 modules under an optimal scale-free topology model fit with a soft threshold of 8 (Supplementary Fig. 3, A-E). For the top modules correlating with CD8 + T cell infiltration (module-trait relationship, green and brown modules), R^2^ of 0.38 and 0.36 were achieved (p-value < 0.05, Supplementary Fig. 3A). With in those modules, 47 (green module) and 125 (brown module) genes were identified respectively. We then extracted all genes from those modules and applied Lasso regression model to select gene features associated with tumor infiltration status. From that, a 70-gene transcription signature was identified within the MMRd-EC tumor regions, which significantly correlated with CD8 + T-cell infiltration status (Fig. 1E). Gene ontology GO-based functional annotation illustrated a tight connection of infiltration levels with interferon signaling, antigen presentation, phagocytosis, TCR signaling and lymphocyte regulation (Fig. 1F). This signature perfectly partitioned MMRd ECs into three immune subgroups (Fig. 1E): the hot group showed upregulation of the top 10 pathways (Fig. 1F); the cold group showed completely opposite patterns; and the intermediate group showed partially upregulated patterns. We then narrowed down genes using a two-step lasso regression, which resulted in 14 core genes distinguishing MMRd tumors into predefined subgroups (Fig. 1G) [27–29]. Of those patients included in the DSP profiling, we compared the density of CD8 + TILs across the three immune subgroups stratified by the 14-gene signature and observed significant intergroup differences, with hot tumors having the highest density of CD8 + TILs compared to the other two groups (p = 0.0037, Fig. 1H). Nevertheless, there was no significant association between the expression pattern and other clinicopathological features based on cohort 1 samples (Table 1). We then tentatively explored the relationship of the 14-gene signature with the external TCGA database containing 158 MSI-H EC patients, and interestingly, we observed that cold tumors were significantly associated with a higher pathological grade (p = 0.0158) and a more advanced disease stage (p = 0.0036, Table 1). Although a high grade and late stage are known to be associated with poor prognosis, our 14-gene signature failed to stratify MMRd EC patients with regard to their EFS based on both the internal cohort and TCGA-UCEC data (Supplementary Fig. 4). Of these 14 genes, DNMT3A was the only gene upregulated in cold tumors but downregulated in hot tumors, while the remaining genes were consistently upregulated in hot tumors (Fig. 2). Similarly, correlation analysis demonstrated that DNMT3A was the only gene negatively correlated with CD8 + T-cell infiltration, while the others all showed a positive correlation with the density of CD8 + TILs (Supplementary Fig. 5).

**Table 1:** Association between immune classification and clinicopathological characteristics of MMRd/MSI tumors

	MMRd Cohort 1				P value	TCGA-UCEC MSI Cohort				P value
	ALL	HOT	INTERMEDIATE	COLD		ALL	HOT	INTERMEDIATE	COLD	
	n = 45	n = 15	n = 15	n = 15		n = 158	n = 39	n = 64	n = 55	
Age at diagnosis, median (range)	53 (45–83)	55 (48–83)	54 (48–67)	49 (45–63)	0.0691	62 (35–88)	64 (46–84)	63 (35–88)	59 (35–85)	0.1657
Histology subtype										
Endometrioid	43	14 (32.5%)	15 (34.9%)	14 (32.5%)	0.5926	149	34 (22.8%)	63 (42.3%)	52 (34.9%)	0.0571
Nonendometrioid	2	1 (50%)	0	1 (50%)		9	5 (55.5%)	1 (11.1%)	3 (33.3%)	
Grade										
1–2	35	10 (28.6%)	13 (37.1%)	12 (34.3%)	0.4066	58	12 (20.7%)	30 (51.7%)	16 (27.6%)	0.0158
3	10	5 (50%)	2 (20%)	3 (30%)		55	16 (29.1%)	14 (25.5%)	25 (45.5%)	
Stage										
I	36	14 (38.9%)	11 (30.5%)	11 (30.5%)	0.3060 (early stage vs. advanced stage)	120	33 (27.5%)	51 (42.5%)	36 (30%)	0.0036 (early stage vs. advanced stage)
II	2	0	0	2 (100%)		12	0	9 (75%)	3 (25%)	
III	6	1 (16.7%)	3 (50%)	2 (33.3%)		22	4 (18.2%)	4 (18.2%)	14 (63.6%)	
IV	1	0	1 (100%)	0		4	2 (50%)	0	2 (50%)	
Total number of early-stage cases	38	14 (36.8%)	11 (28.9%)	13 (34.2%)		132	33 (25%)	60 (45.5%)	39 (29.5%)	
Total number of advanced-stage cases	7	1 (14.3%)	4 (57.1%)	2 (28.6%)		26	6 (23.1%)	4 (15.4%)	16 (61.5%)

### Spatial profiling of immune infiltration of MMRd ECs according to immune subtypes

**Fig. 3:**
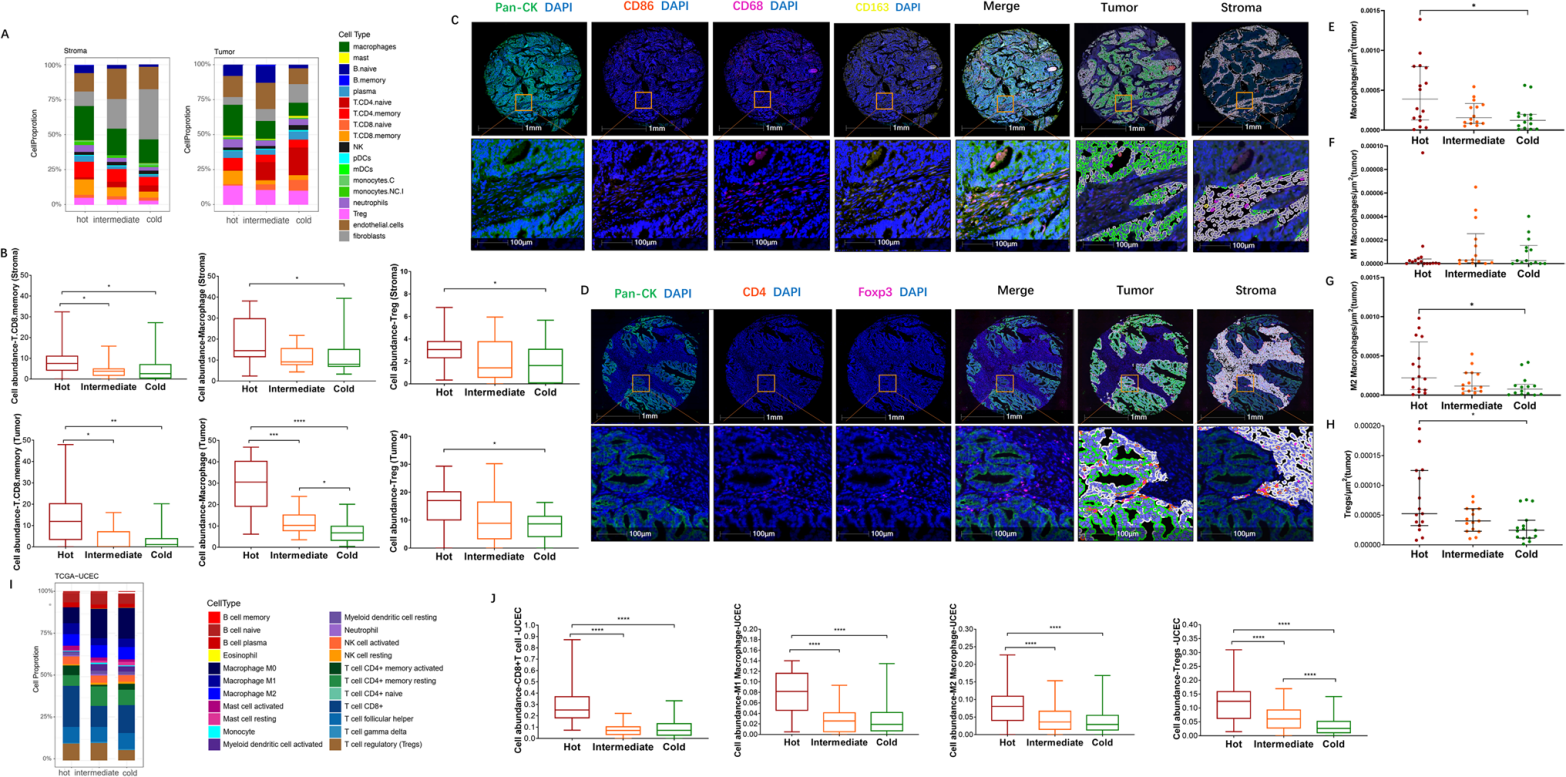
Spatial profiling of immune infiltration of MMRd ECs based on three immune subtypes. (**A**) Identification of 18 types of cells in the tumor region and stromal region by DSP. (**B**) Comparison of the abundance of CD8 + memory T cells, macrophages and Tregs within tumor regions or stromal regions from “hot”, “intermediate” and “cold” tumors in study cohort 1. The Kruskal‒Wallis test with Dunn's multiple comparisons test was used with *p* < 0.05 indicating statistical significance. *, *P* < 0.05, **, *P* < 0.01; ***, *P* < 0.001; ****, *P* < 0.0001. (**C**) Representative images showing mIHC staining of macrophages and digital quantification by HALO software. The bottom row shows zoomed regions corresponding to the orange boxes in the upper row. (**D**) Representative images showing mIHC staining of Tregs and digital quantification by HALO software. The bottom row shows zoomed regions corresponding to the orange boxes in the upper row. (**E**) Comparison of the density of macrophages from hot, intermediate and cold tumors in study cohort 1. The Kruskal‒Wallis test with Dunn's multiple comparisons test was used with *p* < 0.05 indicating statistical significance. *, *P* < 0.05. (**F**) Comparison of the density of M1 macrophages from hot, intermediate and cold tumors in study cohort 1. The Kruskal‒Wallis test with Dunn's multiple comparisons test was used with *p* < 0.05 indicating statistical significance. *, *P* < 0.05. (**G**) Comparison of the density of M2 macrophages from hot, intermediate and cold tumors in study cohort 1. The Kruskal‒Wallis test with Dunn's multiple comparisons test was used with *p* < 0.05 indicating statistical significance. *, *P* < 0.05. (**H**) Comparison of the density of Tregs from hot, intermediate and cold tumors in study cohort 1. The Kruskal‒Wallis test with Dunn's multiple comparisons test was used with *p* < 0.05 indicating statistical significance. *, *P* < 0.05. (**I**) Identification of 22 types of cells in the TCGA MSI EC cohort using the CIBERSORT deconvolution method. (**J**) Comparison of the abundance of CD8 + T cells, M1/M2 macrophages and Tregs from hot, intermediate and cold tumors in the TCGA MSI EC cohort. The Kruskal‒Wallis test with Dunn's multiple comparisons test was used with *p* < 0.05 indicating statistical significance. ****, *P* < 0.0001

Upon classifying EC tumors according to their spatial transcriptional patterns, we then characterized cell type abundance in tumor/stromal areas within the three predefined subtypes (Fig. 3A). Using DSP-deconvoluted data, consistent with the results of CD8 IHC staining, hot tumors had a significantly higher abundance of CD8 + T cells in both tumor and stromal areas than the other two groups, which was also accompanied by the simultaneous upregulation of macrophages and Tregs (Fig. 3B). We then conducted mIHC on these TMAs to evaluate the abundance of macrophages and Tregs at the protein level. As demonstrated, macrophages were defined as CD68 + ; M1-like macrophages were defined as CD68 + CD86 + ; M2-like macrophages were defined as CD68 + CD163 + ; and Tregs were characterized by the copresence of CD4 and FoxP3 (Fig. 3 C/D). Digital quantification showed that in the tumor area, macrophages (Fig. 3E), including M2-like macrophages (Fig. 3F) but not M1-like macrophages (Fig. 3G), were significantly upregulated in hot tumors compared to cold tumors. Similarly, in the tumor area, hot tumors also exhibited significantly higher infiltration of Tregs than cold tumors (Fig. 3H). However, stromal analysis did not give the same conclusion with statistical significance between hot tumors and cold tumors (Supplementary Fig. 6). Overall, the mIHC results further consolidated our findings derived from DSP analysis with tumor-infiltrated macrophages and Tregs present in the transcriptomic-defined hot tumors. To further strengthen our findings, we analyzed the cell components of MMRd/MSI-high ECs from the TCGA cohort (TCGA-UCEC) using the CIBERSORT algorithm (Fig. 3 I/J). Consistent with data from our study cohorts, external data showed a significantly higher abundance of CD8 + TILs, M2 macrophages and Tregs in hot tumors than in cold tumors. The discordance of M1-like macrophage ratios between mIHC data and TCGA data may be related to the larger sample size of the TCGA cohort and bulk data analysis.

**Fig. 4:**
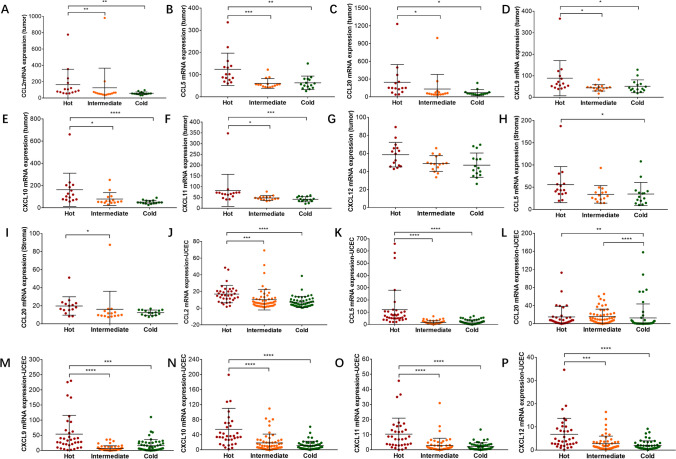
Analysis of the expression of chemokines according to the three immune subtypes**.** (**A**-**G**) Comparison of the expression of CCL2, CCL5, CCL20, CXCL9, CXCL10, CXCL11 and CXCL12 in the tumor region from “hot”, “intermediate” and “cold” tumors in study cohort 1. The Kruskal‒Wallis test with Dunn's multiple comparisons test was used with *p* < 0.05 indicating statistical significance. *, *P* < 0.05, **, *P* < 0.01; ***, *P* < 0.001; ****, *P* < 0.0001. (**H**-**I**) Comparison of the expression of CCL5 and CCL20 in the stromal region from hot, intermediate and cold tumors in study cohort 1. The Kruskal‒Wallis test with Dunn's multiple comparisons test was used with *p* < 0.05 indicating statistical significance. *, *P* < 0.05. (**J**-**P**) Comparison of the expression of CCL2, CCL5, CCL20, CXCL9, CXCL10, CXCL11 and CXCL12 in hot, intermediate and cold tumors in the TCGA MSI EC cohort. The Kruskal‒Wallis test with Dunn's multiple comparisons test was used with *p* < 0.05 indicating statistical significance. **, *P* < 0.01; ***, *P* < 0.001; ****, *P* < 0.0001

Since the migration of immune cells is regulated by chemokines, which may potentially correlate with the varying CD8 + T-cell infiltration status of the three immune subgroups, we analyzed the expression of a series of chemokines in these DSP samples. In tumor regions, the expression levels of *CCL2*, *CCL5*, *CCL20*, *CXCL9*, *CXCL10*, *CXCL11* and *CXCL12* were generally higher in hot tumors than in the other two groups (Fig. 4 A-G). In contrast, in the stromal area, only two chemokines (*CCL5* and *CCL20*) were upregulated in hot tumors (Fig. 4H-I). We then sought to validate this by using TGCA-UCEC data and found that compared with the other two groups, hot tumors harbored the highest expression levels of *CCL2*, *CCL5*, *CCL20*, *CXCL9*, *CXCL10*, *CXCL11* and *CXCL12* (Fig. 4 J-P). Overall, these results suggested a probable chemokine-mediated recruitment of specific immune cells associated with our gene signature-defined immune subtypes.

### HLA class I and DNMT3A as biomarkers for classifying cold and hot MMRd-EC tumors

**Fig. 5:**
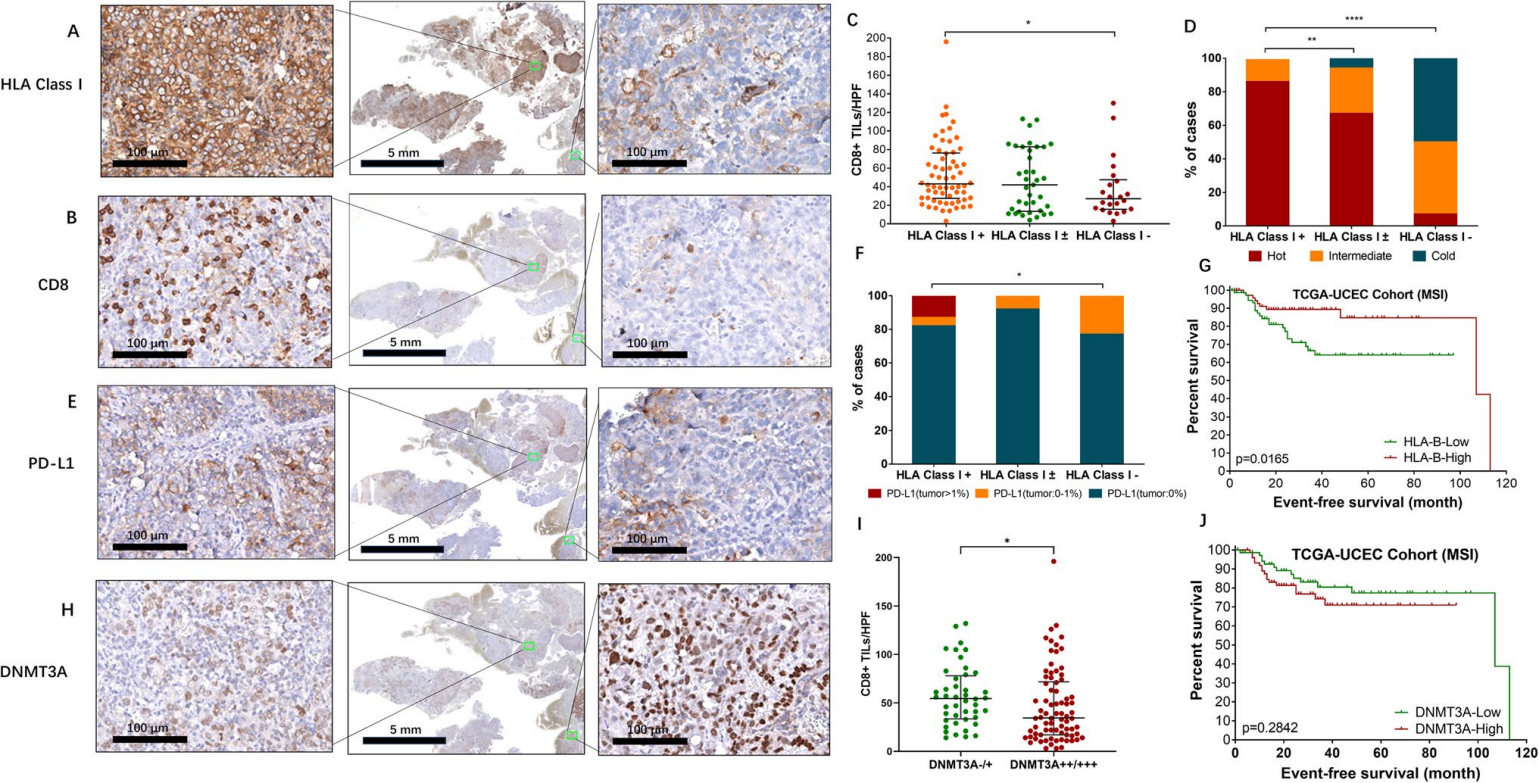
Validation of HLA Class I and DNMT3A as biomarkers for classifying cold and hot MMRd-EC tumors. (**A**) Representative images showing the expression of HLA class I in a full section. The left and right images show details of the regions indicated by green boxes in the middle image. Magnification: 5 times (middle) and 200 times (left and right). (**B**) Representative images showing the expression of CD8 in the full section. The left and right images show details of the regions indicated by green boxes in the middle image. Magnification: 5 times (middle) and 200 times (left and right). (**C**) Comparison of the density of CD8 + TILs from “HLA Class I + ”, “HLA Class I ± ” and “HLA Class I-” tumors. The Kruskal‒Wallis test with Dunn's multiple comparisons test was used, with *p* < 0.05 indicating statistical significance. *, *P* < 0.05. (**D**) Comparison of the proportion of “hot”, “intermediate” and “cold” tumors according to HLA class I status. Chi-square tests were used, with *p* < 0.05 indicating statistical significance. **, *P* < 0.01; ****, *P* < 0.0001. (**E**) Representative images showing the expression of PD-L1 in the full section. The left and right images show details of the regions indicated by green boxes in the middle image. Magnification: 5 times (middle) and 200 times (left and right). (**F**) Comparison of the proportion of tumors with different PD-L1 staining patterns according to HLA class I status. Chi-square tests were used, with *p* < 0.05 indicating statistical significance. *, *P* < 0.05. (**G**) Prognostic impact of HLA-B on MSI ECs in the TCGA cohort. The log-rank test was applied, with *p* < 0.05 indicating statistical significance. (**H**) Representative images showing the expression of DNMT3A in the full section. The left and right images show details of the regions indicated by green boxes in the middle image. Magnification: 5 times (middle) and 200 times (left and right). (**I**) Comparison of the density of CD8 + TILs from HLA Class I + , HLA Class I ± and HLA Class I- tumors. The Kruskal‒Wallis test with Dunn's multiple comparisons test was used, with *p* < 0.05 indicating statistical significance. *, *P* < 0.05. (**J**) Prognostic impact of DNMT3A on MSI ECs in the TCGA cohort. The log-rank test was applied, with *p* < 0.05 indicating statistical significance

Since our DSP data highlighted potential connections of HLA class I molecule/DNMT3A functioning with CD8 + T-cell infiltration, we then validated the result at the protein level. Upon characterization of all MMRd tumors from cohort 1 and cohort 2 by IHC, a strong positive correlation between HLA class I expression and CD8 + TILs was observed, with 50.4% showing HLA class I positivity; 31.4% of tumors harbored subclonal loss; and 18.2% had negative expression. Tumor areas with strong expression of HLA class I displayed high infiltration of CD8 + TILs, while weaker HLA class I-expressing areas showed a lower density of CD8 + TILs (Fig. 5 A/B). Across these MMRd cases, HLA class I-positive tumors had significantly higher CD8 + T-cell infiltration than HLA class I-negative tumors (p = 0.0131, Fig. 5C). The proportion of HLA class I-positive tumors was significantly higher in the hot tumors defined by the 14-gene signature (p = 0.0001, Fig. 5D), supporting our previous DSP data with HLA-B positively correlated with CD8 + TILs (r = 0.3261, p = 0.0288, Fig. 5D). Notably, all PD-L1-positive (TC) cases were also HLA class I-positive (Fig. 5E/F), suggesting that HLA class I positivity may serve as a sensitive biomarker for anti-PD-1/PD-L1 therapy response. Interestingly, although individual gene-based survival analysis was not indicative of any prognostic value in our cohort 1 study, TCGA data indicated that high expression of HLA-B was associated with better EFS in MSI ECs, implying a likely prognostic role of HLA-B in predicting MSI EC patient survival (p = 0.0165, Fig. 5G).

For DNMT3A, although IHC data exhibited intrapatient heterogeneity in some tumors with different intensities in different tumor areas (Fig. 5H), a general trend of high CD8 + TILs associated with weak staining of DNMT3A was observed and vice versa. We then compared the density of CD8 + TILs according to DNMT3A staining in ECs from our study cohorts and found that tumors with moderate/strong DNMT3A expression had a significantly lower density of CD8 + TILs than tumors with negative/weak expression of DNMT3A based on 123 MMRd ECs (p = 0.0183, Fig. 5I). In addition, although not statistically significant, survival analysis of TCGA MSI-EC data exhibited a trend indicative of the prognostic value of *DNMT3A*, with higher expression associated with worse EFS (Fig. 5J). However, regardless of the MSI status, high expression of *DNMT3A* was significantly associated with poorer EFS in the TCGA-EC cohort (Supplementary Fig. 7). Due to the negative association between HLA class I and DNMT3A, we then analyzed the mRNA expression of *HLA-B* and *DNMT3A* across cohort 1 samples and found that in the tumor area, there was a negative correlation between the two genes (r = -0.4649, p = 0.0013 Supplementary Fig. 8A). This association was also observed in the TCGA cohort, where MSI-ECs showed a negative correlation between *HLA-B* and *DNMT3A* (r = -0.2221, p = 0.0052 Supplementary Fig. 8B). Since chemokines play an important role in recruiting T cells [30–34], we also analyzed the correlation of *DNMT3A* mRNA expression and chemokines detected in DSP. A negative correlation between *DNMT3A* expression and *CCL5* expression was observed (r = -0.3805, p = 0.0099 Supplementary Fig. 8C). We then evaluated the correlation between *DNMT3A* and *CCL5* receptors and found that their expression was also negatively correlated within the tumor regions (Supplementary Fig. 8D/E). Moreover, these findings could also be validated using TCGA MSI-EC data (Supplementary Fig. 8F-H). Taken together, these results suggested that the negative correlation between DNMT3A and CD8 + T-cell infiltration is associated with the downregulation of HLA class I and *CCL5*.

## Discussion

MMRd tumors are known to carry high loads of neoantigens, which is believed to be a key biological cause of T-cell recruitment and to trigger local immunogenic reactions within the TME. These mechanisms generate a predisposition to checkpoint inhibitor therapy response. Nevertheless, cumulative evidence suggests that lymphocyte infiltration levels vary significantly across cancer types, a major limiting factor in acquiring an optimal response in patients receiving ICI therapies [9, 17, 35]. In addition, MMRd tumors are more sensitive to activated CD8 + T cells and thus respond better to ICI therapies than MMRp tumors with the same TMB load [17]. Therefore, in addition to TMB, other mechanisms may drive CD8 + T-cell infiltration and response to ICI treatment.

Since a subset of MMRd-ECs exhibit a T-cell deprivation pattern, we investigated MMRd-EC from a TME perspective. Using a high-plex spatial transcriptomic approach, a highly resolved tumor-immune landscape was established. This proved that MMRd-EC is a heterogeneous disease and led to the discovery of three immune subtypes at subhistological levels specifically within epithelium-enriched regions. A 70-gene transcription program was identified stratifying patients into hot, intermediate and cold groups, which correlated with CD8 + T-cell infiltration, and this immune-phenotypic effect was mainly mediated by altered antigen presentation, T-cell trafficking, and TCR signaling. Further extraction resulted in a 14-gene core signature driving the transition of inflammation status from cold to hot with a corresponding increase in CD8 + T-cell infiltration.

Spatial deconvolution-based immune cell typing also supported our finding that the hot subtype had the highest infiltration of CD8 + TILs, thereby promoting local cancer cell killing [36]. However, evidence from both DSP-based deconvolution and mIHC suggested that antitumor effects may partially be neutralized by increasing levels of M2-like macrophages and Tregs found in hot tumors [36–39]. This also explains the limited clinical benefits in both our study cohorts and the TCGA cohort, where there was no difference in the prognosis of the subgroup with the highly inflamed TME compared to the other two MMRd-EC subgroups. Presumably, this immune normalization may be attributed to the dual role of chemokines that recruit both antitumor immune cells and protumor immune cells [32], as in our spatial profiling, hot tumors exhibited the highest expression levels of *CCL5*, *CXCL9*, *CXCL10*, *CXCL11*, *CCL2*, *CXCL12* and *CCL20*, wherein CD8 + T cells can be recruited by CCL5, CXCL9, CXCL10 and CXCL11, but CCL5 can also recruit tumor-associated macrophages, which can also be recruited by CCL2 and CXCL12 [40–44], while Tregs are recruited by CCL20 [43, 45, 46].

Of the 14 key genes identified, HLA class I genes were major contributors to immune subtype classification; therefore, we subsequently assessed key molecules. Indeed, the positive correlation between HLA class I and CD8 + TILs was observed at the protein level based on 123 MMRd-EC patients, and TCGA-MSI cohort analysis also revealed the prognostic value of this marker. Functional HLA class I plays key roles in presenting tumor-associated peptides to CD8 + T cells, explaining their regulatory synergy in a hot TME [47, 48], whereas loss of HLA class I expression impairs recognition of tumor-associated antigens by CD8 + T cells, a key mechanism for immune evasion, causing tumor progression and insensitivity to ICI therapy [48–51]. Previous studies have shown that loss of NOD-Like Receptor C5 (NLRC5), a key transcription factor, regulates HLA class I pathway gene transcription in multiple cancers and causes reduced expression of its target genes, such as HLA class I genes (*HLA-A*, *B*, *C*), *β2M* and *TAP* [52–54]. This is consistent with our observation that *NLRC5*, *HLA-A*, *HLA-B*, *β2M* and *TAPBP* were downregulated in cold tumors (Fig. 1E), suggesting a coregulatory network functioning via *NLRC5-*mediated HLA molecule expression. Moreover, since the level of the HLA class I complex and its relative components within the pathway can be induced by type I/II interferon, our results also demonstrated that type I/II interferon pathways and key signaling components such as *STAT1*, *STAT2* and *IRF9* were upregulated in hot tumors [55]. Interestingly, our results showed that ECs expressing PD-L1 in ≥ 1% of tumor cells were more likely to have fully intact HLA class I than PD-L1-negative ECs, a finding consistent with a previous report by Friedman et al. [24]. Collectively, HLA class I could serve as a promising biomarker for MMRd-EC candidate selection in ICI therapies. We also noted a negative correlation between DNMT3A expression and CD8 + T-cell infiltration at both the mRNA and protein levels and a tendency toward worse prognosis with high DNMT3A expression. In addition, we found that high expression of *DNMT3A* was associated with poorer EFS in ECs based on the TCGA-EC cohort (Supplementary Fig. 5). Functioning through its DNA methyltransferase activity, DNMT3A plays a critical role in epigenetic regulation [56]. Overexpression of DNMT3A is associated with oncogenesis in multiple cancers via epigenetic silencing of pivotal tumor suppressor genes and distortion of T-cell function [56–59]. The antagonistic modulation between DNMT3A and HLA class I in our study and the TCGA ECs implies that DNMT3A may be involved in epigenetic regulation of HLA class I. Previous studies support the notion that inhibition of DNA methyltransferases causes elevated HLA class I expression in several HLA class I low cancer cell lines [60–63], and functional restoration by DNA methyltransferase inhibitors is associated with transcriptomic upregulation of genes in the HLA class I antigen presentation pathway [63, 64]. We hypothesized that DNMT3A may also suppress chemokines to discharge lymphocytes from evolving tumor regions. Our data at spatial transcriptomic and proteomic levels revealed negative regulatory feedback between DNMT3A and CCL5 as well as its receptors (CCR1 and CCR5), resulting in dynamic alteration of CD8 + TILs in a subset of MMRd-ECs. Our study may provide preliminary insight linking DNMT3A to CCL5 in ECs, and thus, work needs to be extended to address the mechanism by which DNMT3A regulates the expression of CCL5 in ECs. In summary, our biomarker-driven exploration uncovered a new direction in exploring the DNMT3A-mediated function in regulating HLA class I and chemokines that drive CD8 + T-cell infiltration in MMRd-ECs.

In conclusion, our comprehensive spatial profiling resolved the interpatient heterogeneity of MMRd-ECs, underscoring a plausible patient stratification approach that is of clinical importance for ICI therapies. The 14-gene signature and MHC class I and CD8 IHC may be used as biomarkers to select candidates for ICI treatment. In addition, deconvoluting the underlying mechanism of HLA class I/DNMT3A/CD8 T-cell modulation in MMRd-ECs will accelerate biomarker translation and the development of combination treatment regimens for advanced EC.

### Supplementary Information

Below is the link to the electronic supplementary material.

Fig. S1 Representative IHC staining patterns of HLA Class I. (PNG 4543 kb)

High Resolution (TIF 13061 kb)

Fig. S2 Representative IHC staining patterns of DNMT3A. (PNG 2331 kb)

High Resolution (TIF 6715 kb)

Fig. S3 Modules identified via WGCNA (A) Five modules (color annotated on the left column) identified through WGNCA using DSP-CTA data on tumor-enriched regions and their module-trait relationships. Colors show module-trait correlation with red indicating positive correlation with CD8+ T cell infiltration and green showing the opposite (R2 ranging from -1 to 1). (B) Cluster dendrograms of genes associated with each module. (C) Eigen adjacency heatmap between modules. (D) ROI-wise clustering dendrogram. (E) Network topology with various soft thresholds. (left) Scale-free fit (y-axis) and soft-thresholding power (x-axis). (right) Mean connectivity between networks (y-axis) and soft-thresholding power (x-axis).(PNG 447 kb)

High Resolution (TIF 2991 kb)

Fig. S4 Survival analysis according to three immune subgroups. (A) Comparison of the prognosis of the three immune subgroups in the study cohort. (B) Comparison of the prognosis of the three immune subgroups in the TCGA MSI EC cohort. The log-rank test was applied, with p < 0.05 indicating statistical significance.(PNG 124 kb)

High Resolution (TIF 467 kb)

Fig. S5 Expression association of the 14 genes with the density of CD8+TILs. HLA-B, PAPR9, TNFRSF14, IFI16, HLA-DMA, TYMP, TAPBP, IRF9, FCER1G, CD53, CD4, WIPF1, RAC2 and DNMT3A are shown in separate panels. Abbreviations: HPF, high-power field. The Pearson correlation coefficient (R^2^) and p values are shown.(PNG 126 kb)

High Resolution (TIF 1117 kb)

Fig. S6 Immune infiltration profiling in the stromal region of MMRd ECs according to their immune subtypes using mIHC. (A) Comparison of the density of macrophages from “hot”, “intermediate” and “cold” tumors in study cohort 1. (B) Comparison of the density of M1 macrophages from hot, intermediate and cold tumors in study cohort 1. (C) Comparison of the density of M2 macrophages from hot, intermediate and cold tumors in study cohort 1. (D) Comparison of the density of Tregs from “hot”, “intermediate” and “cold” tumors in study cohort 1. The Kruskal‒Wallis test with Dunn's multiple comparisons test was used with p < 0.05 indicating statistical significance. *, P < 0.05.(PNG 100 kb)

High Resolution (TIF 579 kb)

Fig. S7 Prognostic impact of DNMT3A on ECs in the TCGA cohort. The log-rank test was applied, with p < 0.05 indicating statistical significance. (PNG 197 kb)

High Resolution (TIF 224 kb)

Fig. S8 Correlation analysis between DNMT3A and HLA-B, CCL5, CCR1, and CCR5 in the study cohort and the TCGA MSI EC cohort. Pearson correlation analysis was used, and p values are shown. Abbreviations: MSI, microsatellite instability; HPF, high-power field. (PNG 240 kb)

High Resolution (TIF 1280 kb)

Table S1 (DOCX 17 kb)

Table S2 (DOCX 19 kb)

Table S3 (DOCX 21 kb)

Table S4 (XLSX 30 kb)

Table S5 (DOCX 15 kb)

## Data Availability

All data generated or analyzed during this study are included in this published article and its supplementary information files.
