# Supplementary figures and images for: High-plex spatial transcriptomic profiling reveals distinct immune components and the HLA class I/DNMT3A/CD8 modulatory axis in mismatch repair-deficient endometrial cancer
Source: Cell Oncol (Dordr). 2023 Oct 17;47(2):573–85. doi: 10.1007/s13402-023-00885-8 (PMC11090934; doi:10.1007/s13402-023-00885-8)

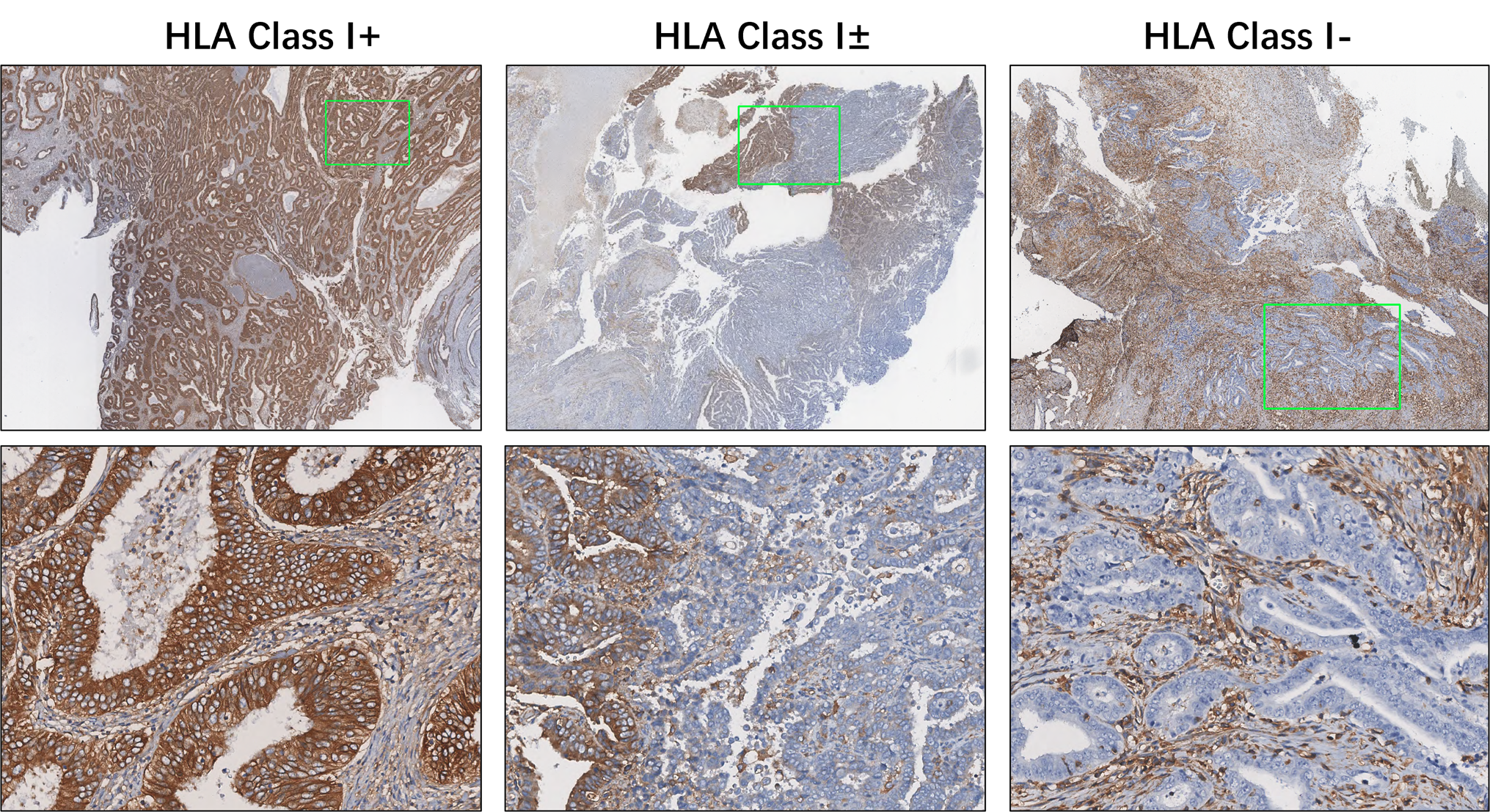

Supplement: Supplementary file 1 — Representative IHC staining patterns of HLA Class I. (PNG 4543 kb) [file 13402_2023_885_Fig6_ESM.png]

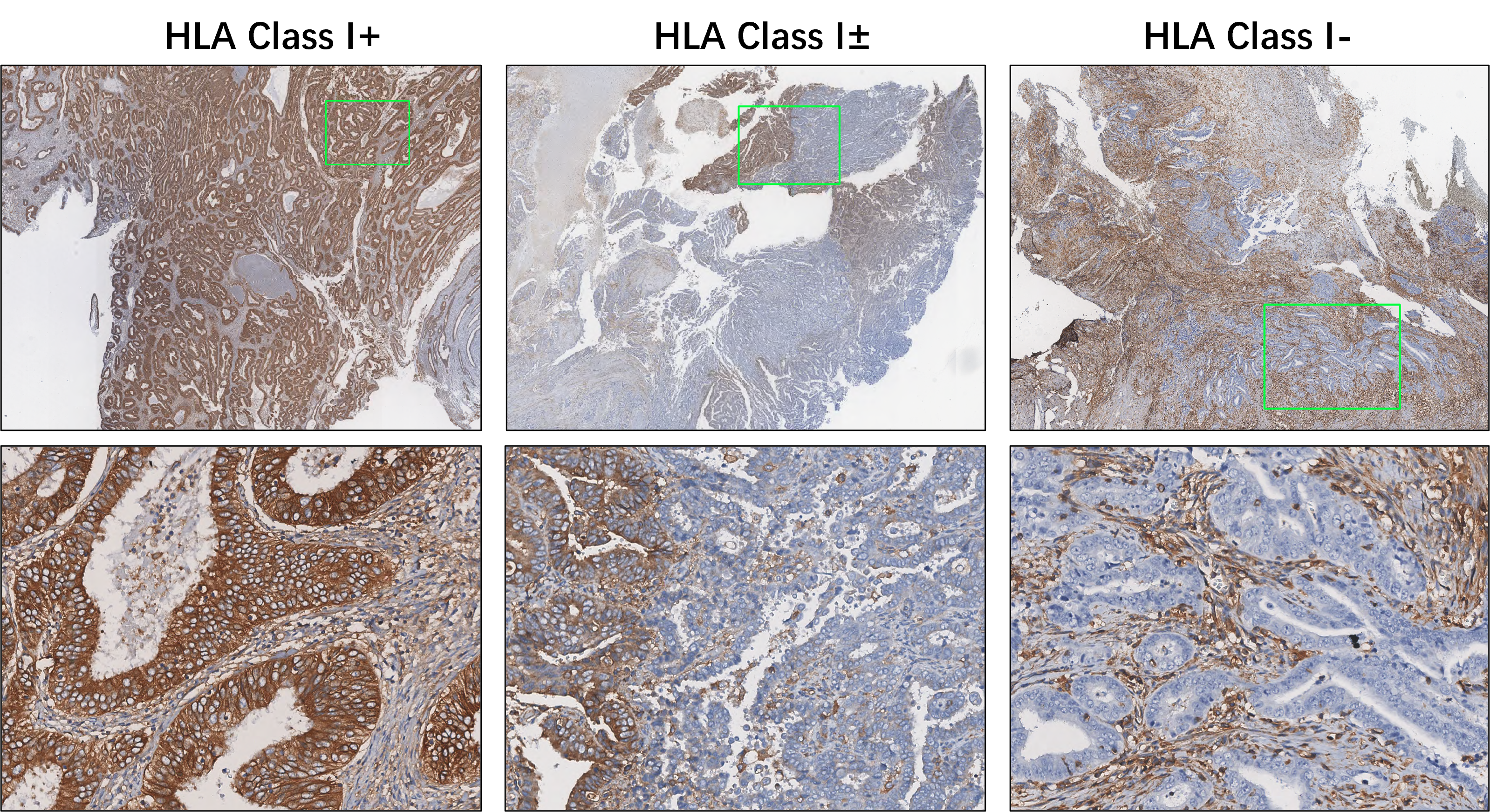

Supplement: Supplementary file 2 — High Resolution (TIF 13061 kb) [file 13402_2023_885_MOESM2_ESM.tif]

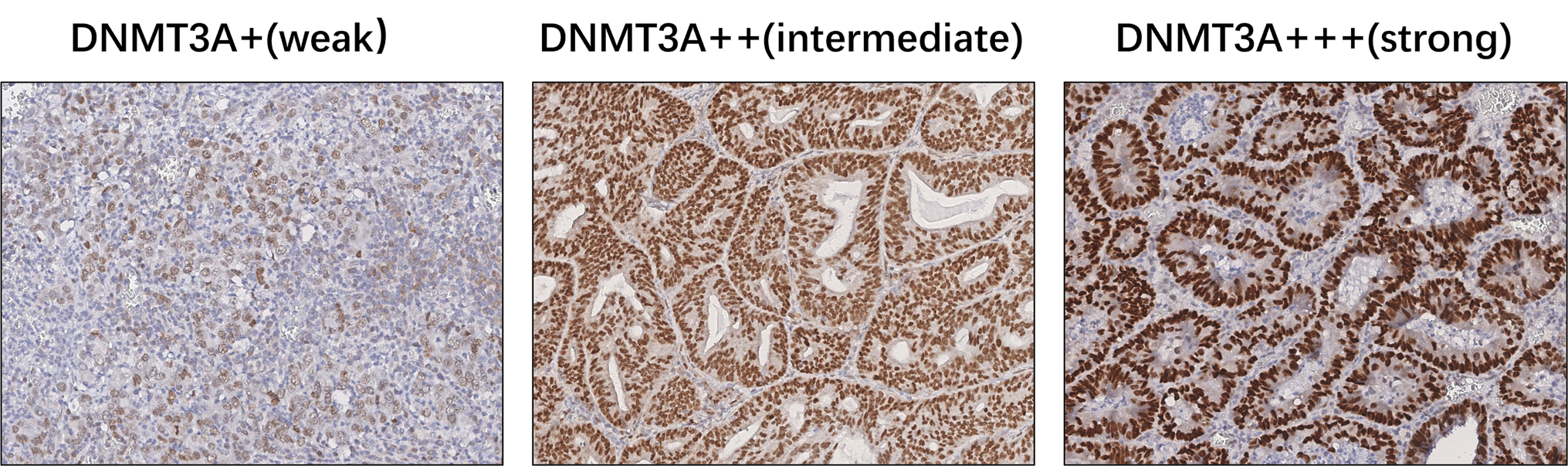

Supplement: Supplementary file 3 — Representative IHC staining patterns of DNMT3A. (PNG 2331 kb) [file 13402_2023_885_Fig7_ESM.png]

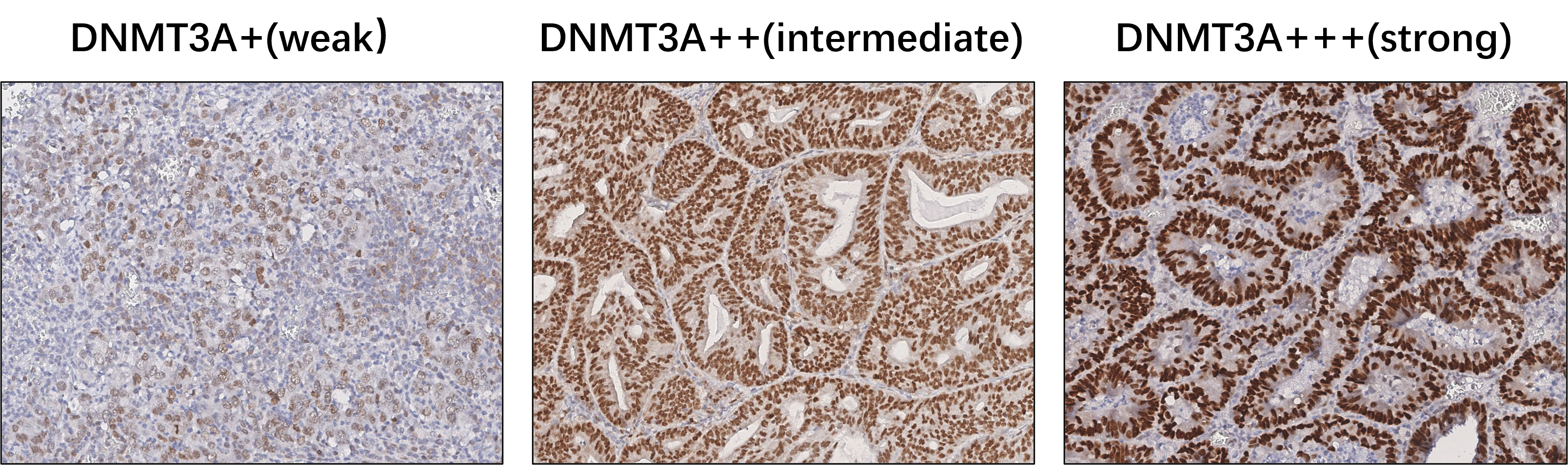

Supplement: Supplementary file 4 — High Resolution (TIF 6715 kb) [file 13402_2023_885_MOESM4_ESM.tif]

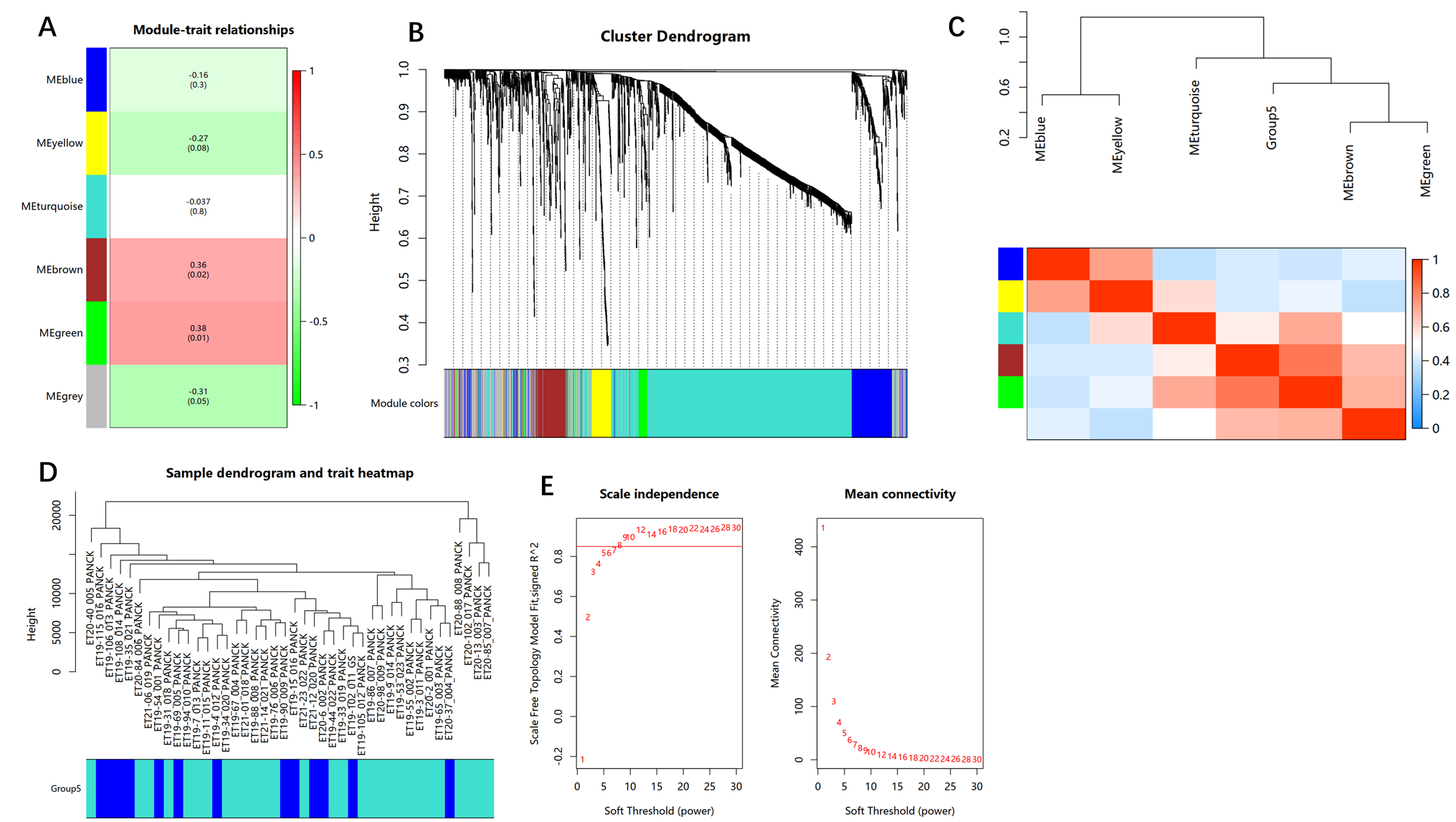

Supplement: Supplementary file 5 — Modules identified via WGCNA (A) Five modules (color annotated on the left column) identified through WGNCA using DSP-CTA data on tumor-enriched regions and their module-trait relationships. Colors show module-trait correlation with red indicating positive correlation with CD8+ T cell infiltration and green showing the opposite (R2 ranging from -1 to 1). (B) Cluster dendrograms of genes associated with each module. (C) Eigen adjacency heatmap between modules. (D) ROI-wise clustering dendrogram. (E) Network topology with various soft thresholds. (left) Scale-free fit (y-axis) and soft-thresholding power (x-axis). (right) Mean connectivity between networks (y-axis) and soft-thresholding power (x-axis).(PNG 447 kb) [file 13402_2023_885_Fig8_ESM.png]

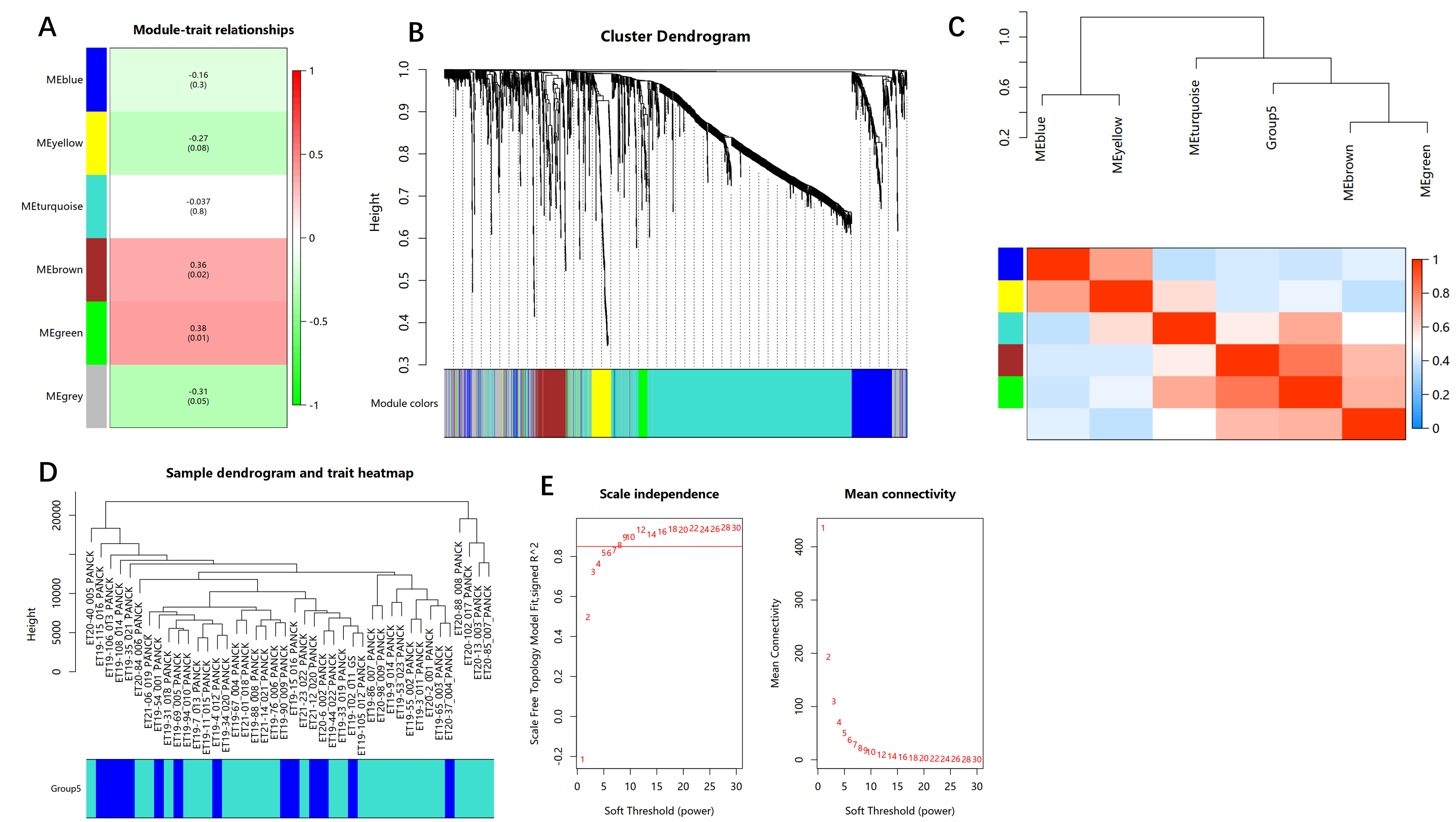

Supplement: Supplementary file 6 — High Resolution (TIF 2991 kb) [file 13402_2023_885_MOESM6_ESM.tif]

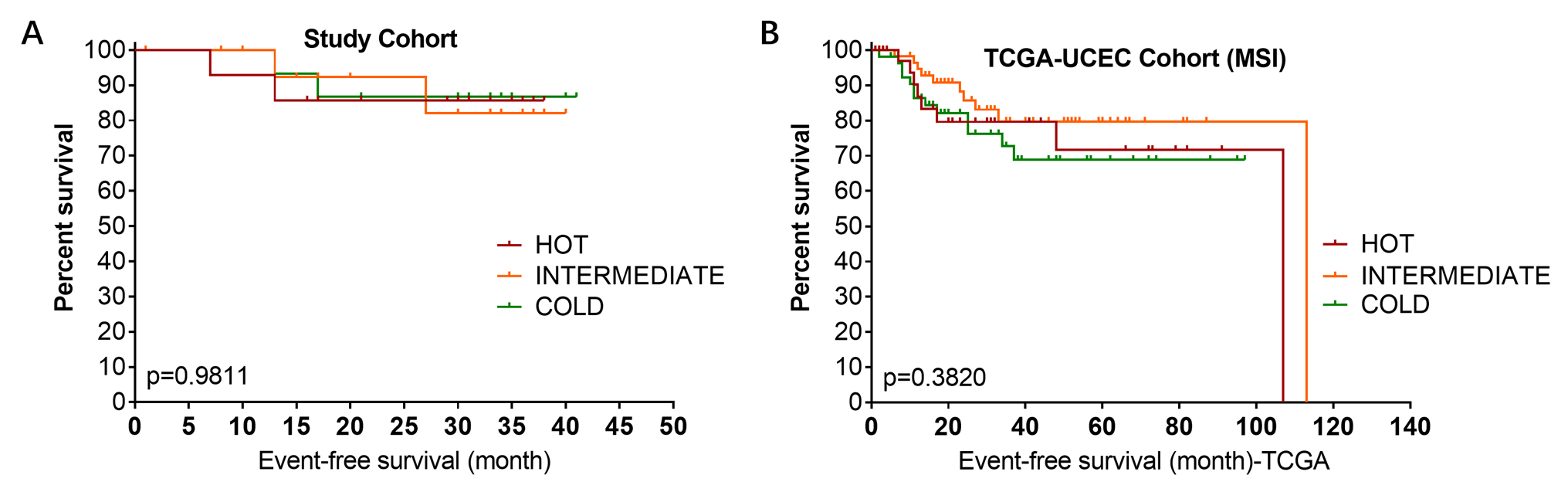

Supplement: Supplementary file 7 — Survival analysis according to three immune subgroups. (A) Comparison of the prognosis of the three immune subgroups in the study cohort. (B) Comparison of the prognosis of the three immune subgroups in the TCGA MSI EC cohort. The log-rank test was applied, with p < 0.05 indicating statistical significance.(PNG 124 kb) [file 13402_2023_885_Fig9_ESM.png]

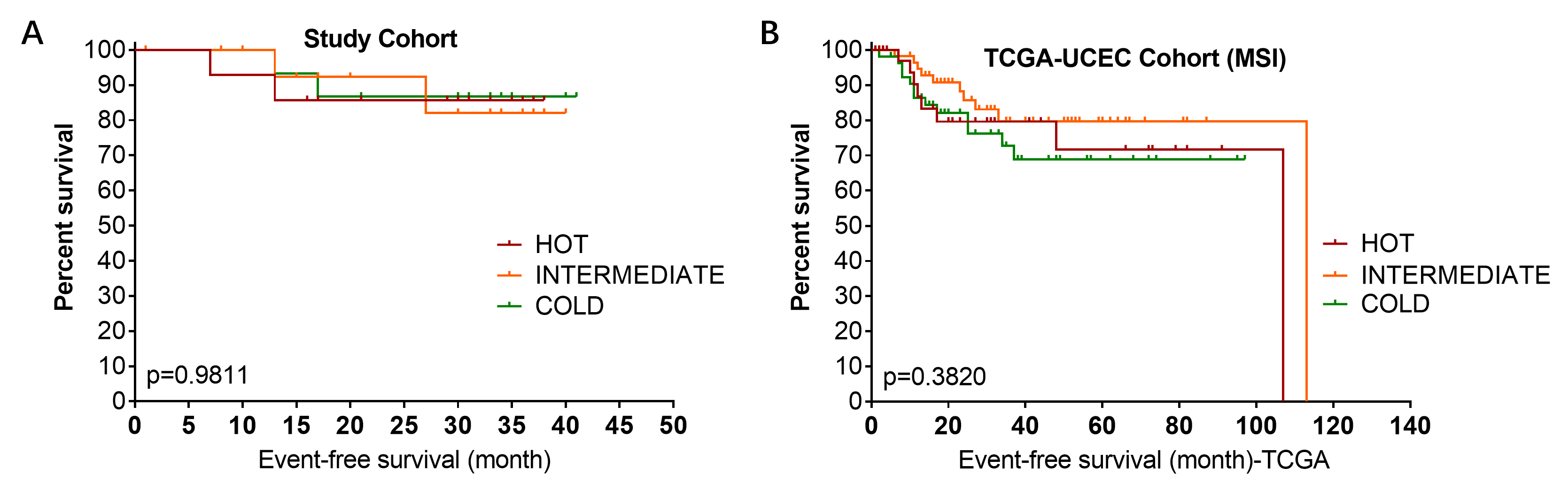

Supplement: Supplementary file 8 — High Resolution (TIF 467 kb) [file 13402_2023_885_MOESM8_ESM.tif]

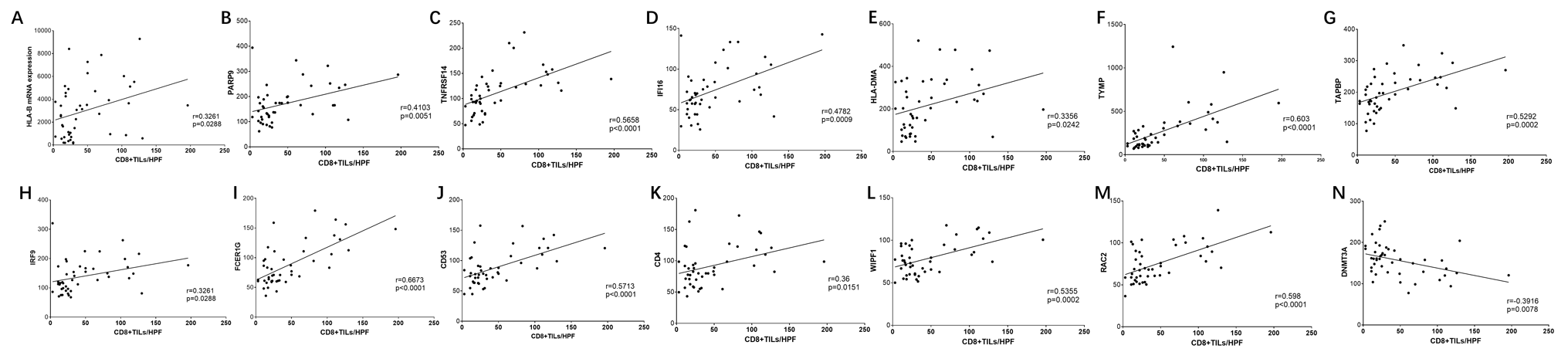

Supplement: Supplementary file 9 — Expression association of the 14 genes with the density of CD8+TILs. HLA-B, PAPR9, TNFRSF14, IFI16, HLA-DMA, TYMP, TAPBP, IRF9, FCER1G, CD53, CD4, WIPF1, RAC2 and DNMT3A are shown in separate panels. Abbreviations: HPF, high-power field. The Pearson correlation coefficient (R2) and p values are shown.(PNG 126 kb) [file 13402_2023_885_Fig10_ESM.png]

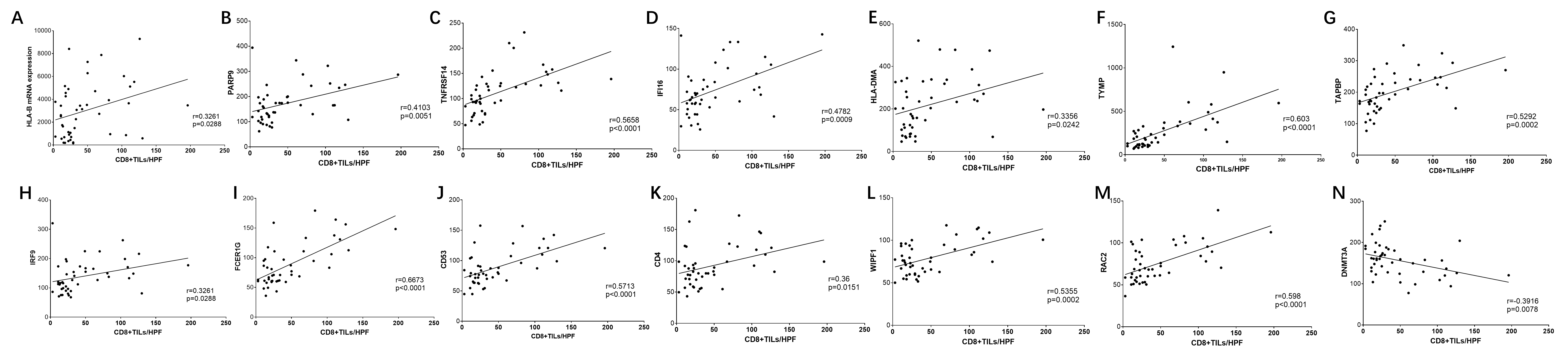

Supplement: Supplementary file 10 — High Resolution (TIF 1117 kb) [file 13402_2023_885_MOESM10_ESM.tif]

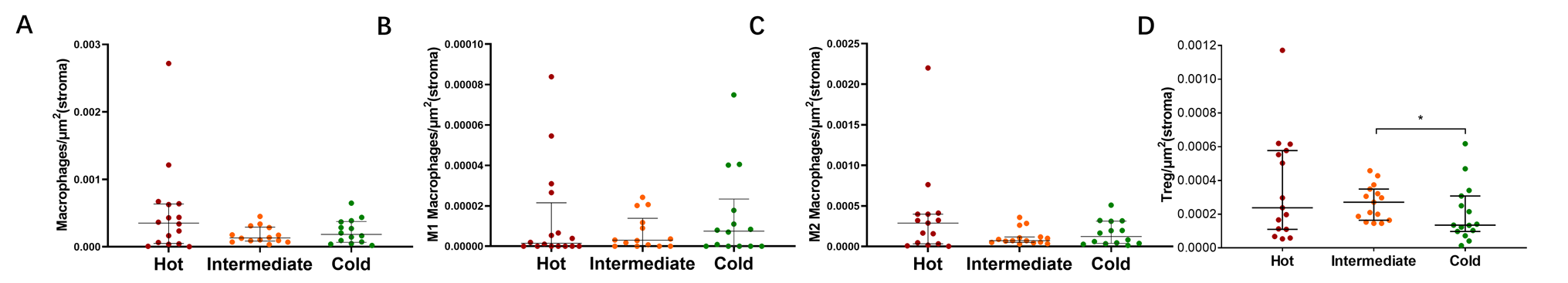

Supplement: Supplementary file 11 — Immune infiltration profiling in the stromal region of MMRd ECs according to their immune subtypes using mIHC. (A) Comparison of the density of macrophages from “hot”, “intermediate” and “cold” tumors in study cohort 1. (B) Comparison of the density of M1 macrophages from hot, intermediate and cold tumors in study cohort 1. (C) Comparison of the density of M2 macrophages from hot, intermediate and cold tumors in study cohort 1. (D) Comparison of the density of Tregs from “hot”, “intermediate” and “cold” tumors in study cohort 1. The Kruskal‒Wallis test with Dunn's multiple comparisons test was used with p < 0.05 indicating statistical significance. *, P < 0.05.(PNG 100 kb) [file 13402_2023_885_Fig11_ESM.png]

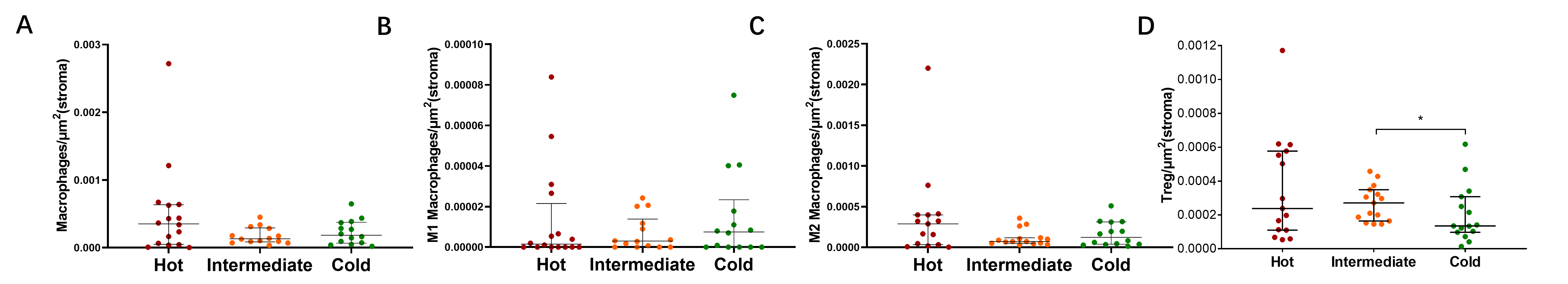

Supplement: Supplementary file 12 — High Resolution (TIF 579 kb) [file 13402_2023_885_MOESM12_ESM.tif]

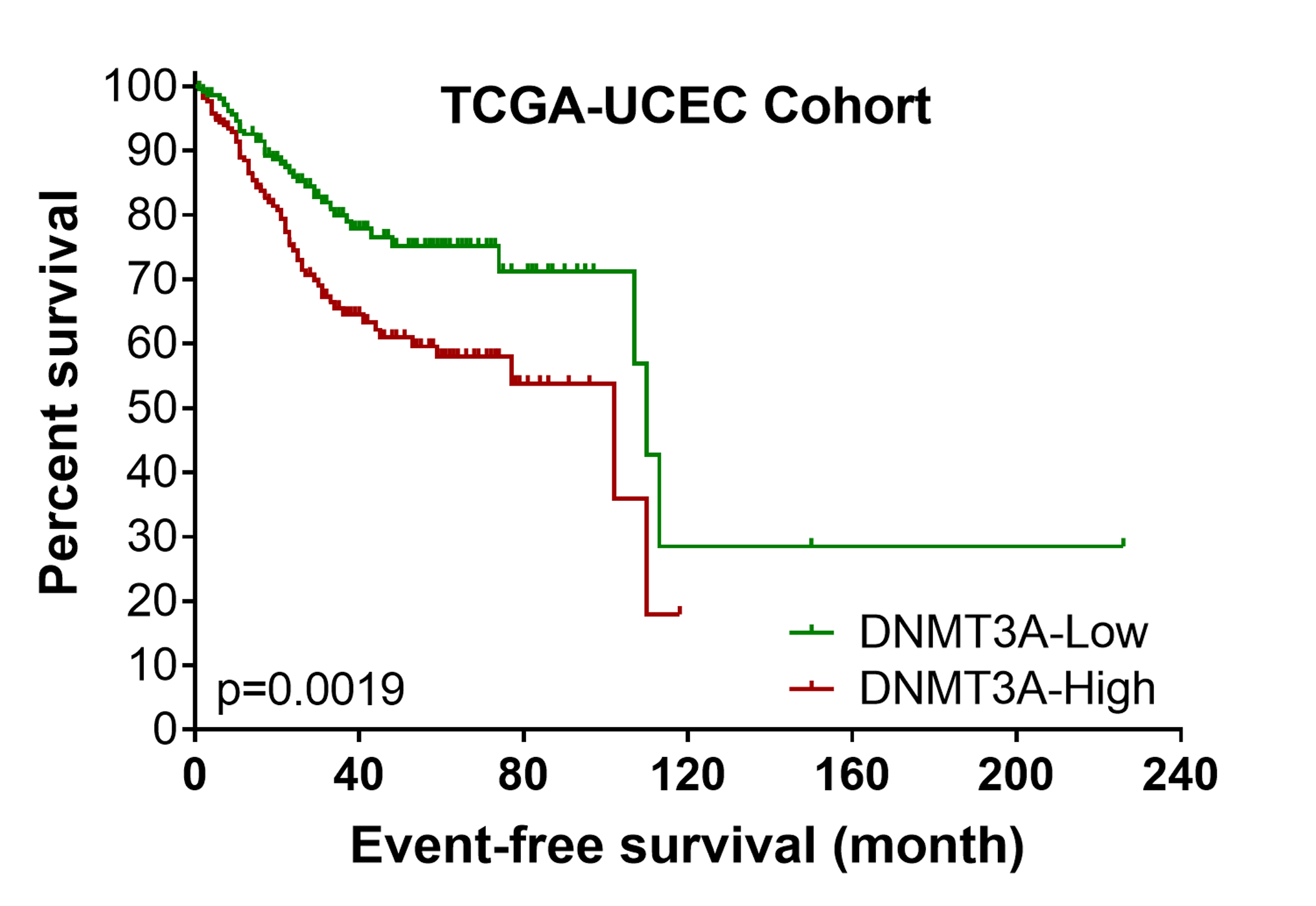

Supplement: Supplementary file 13 — Prognostic impact of DNMT3A on ECs in the TCGA cohort. The log-rank test was applied, with p < 0.05 indicating statistical significance. (PNG 197 kb) [file 13402_2023_885_Fig12_ESM.png]

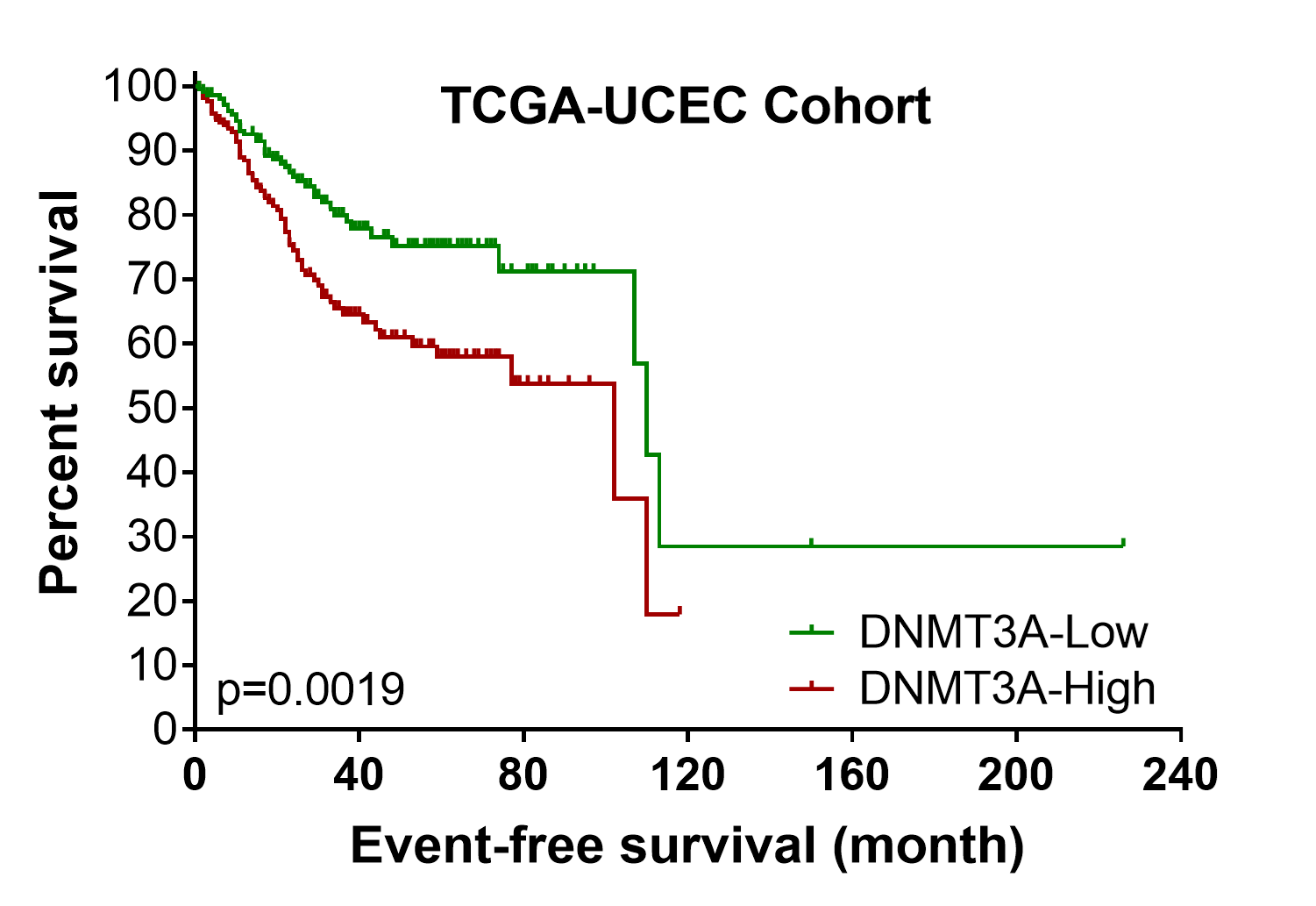

Supplement: Supplementary file 14 — High Resolution (TIF 224 kb) [file 13402_2023_885_MOESM14_ESM.tif]

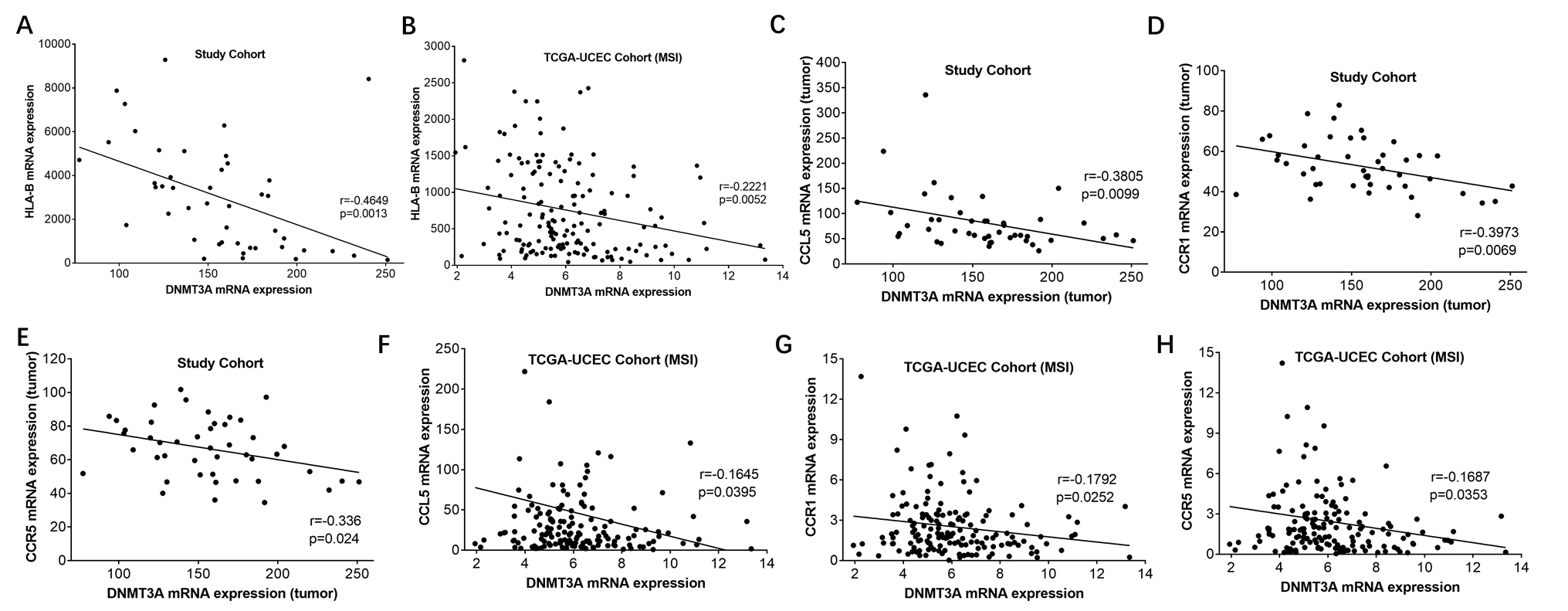

Supplement: Supplementary file 15 — Correlation analysis between DNMT3A and HLA-B, CCL5, CCR1, and CCR5 in the study cohort and the TCGA MSI EC cohort. Pearson correlation analysis was used, and p values are shown. Abbreviations: MSI, microsatellite instability; HPF, high-power field. (PNG 240 kb) [file 13402_2023_885_Fig13_ESM.png]

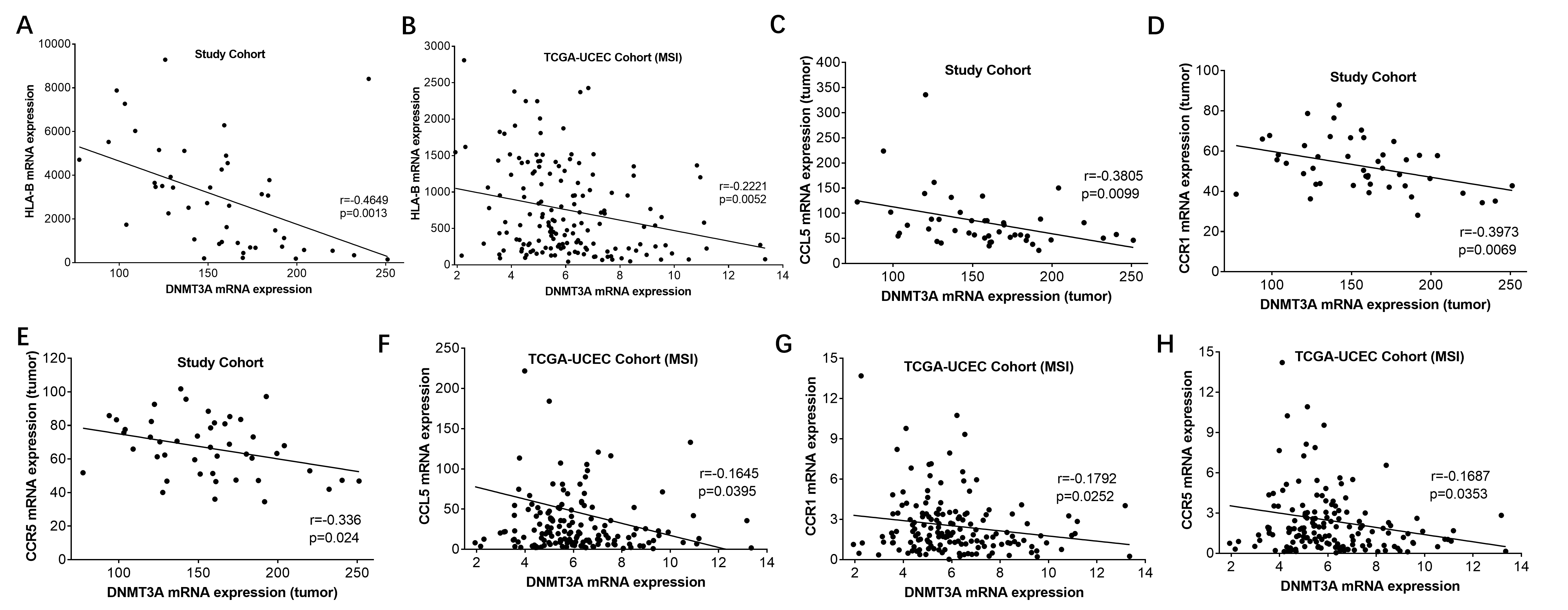

Supplement: Supplementary file 16 — High Resolution (TIF 1280 kb) [file 13402_2023_885_MOESM16_ESM.tif]
