# Supplementary material for: High-plex spatial transcriptomic profiling reveals distinct immune components and the HLA class I/DNMT3A/CD8 modulatory axis in mismatch repair-deficient endometrial cancer
Source: Cell Oncol (Dordr). 2023 Oct 17;47(2):573–85. doi: 10.1007/s13402-023-00885-8 (PMC11090934; doi:10.1007/s13402-023-00885-8)
Supplement: Supplementary file 17 — (DOCX 17 kb) [file 13402_2023_885_MOESM17_ESM.docx]

Supplementary table 1. List of antibodies used in IHC or mIHC.

| Antibodies | Clone | Manufacture | Dilution |
| --- | --- | --- | --- |
| MLH1 | OTI4H4 | ZSGB-BIO | 1:200 |
| MSH2 | RED2 | ZSGB-BIO | 1:400 |
| MSH6 | EP49 | ZSGB-BIO | 1:200 |
| PMS2 | EP51 | ZSGB-BIO | 1:50 |
| CD8 | SP16 | MXB Biotechnologies | prediluted (IHC) |
| PD-L1 | SP142 | ZSGB-BIO | prediluted |
| Pan-CK | C-11 | Abcam | 1:200 |
| CD45 | UCHL1 | CST | 1:400 |
| CD4 | BP6028 | Biolynx | 1:200 |
| FoxP3 | D2W8E | CST | 1:100 |
| CD68 | EPR20545 | Abcam | 1:2000 |
| CD163 | OTI2G12 | Abcam | 1:150 |
| CD86 | E2G8P | CST | 1:200 |
| HLA CLASSⅠABC | EMR8-5 | Abcam | 1:20000 |
| DNMT3A | EPR18455 | Abcam | 1:2000 |
