# Supplementary material for: High-plex spatial transcriptomic profiling reveals distinct immune components and the HLA class I/DNMT3A/CD8 modulatory axis in mismatch repair-deficient endometrial cancer
Source: Cell Oncol (Dordr). 2023 Oct 17;47(2):573–85. doi: 10.1007/s13402-023-00885-8 (PMC11090934; doi:10.1007/s13402-023-00885-8)
Supplement: Supplementary file 18 — (DOCX 19 kb) [file 13402_2023_885_MOESM18_ESM.docx]

|  | **Total**  **n=123** | **MMRd Cohort 1 (DSP, mIHC and IHC)**  **n=45** | **MMRd Cohort 2 (IHC only)**  **n=78** |
| --- | --- | --- | --- |
| **Age at diagnosis, Mean (range)** | 56 (41-83) | 54 (45-83) | 57 (41-76) |
| **Histology subtype** |  |  |  |
| Endometrioid | 118 (95.9%) | 43 (95.6%) | 75 (96.2%) |
| Clear cell | 2 (1.6%) | 1 (0.2%) | 1 (1.3%) |
| Serous | 2 (1.6%) | 0 | 2 (2.5%) |
| Undifferentiated | 1 (0.8%) | 1 (0.2%) | 0 |
| **Grade** |  |  |  |
| 1-2 | 94 (76.4%) | 35 (77.8%) | 59 (75.6%) |
| 3 | 29 (23.6%) | 10 (22.2%) | 19 (24.4%) |
| **Stage** |  |  |  |
| I | 93 (75.6%) | 36 (80.0%) | 57 (73.1%) |
| II | 5 (4.1%) | 2 (4.4%) | 3 (3.8%) |
| III | 23 (18.7%) | 6 (13.3%) | 17 (21.8%) |
| IV | 2 (1.6%) | 1 (2.2%) | 1 (1.3%) |
| **MMRd pattern** |  |  |  |
| Loss of MLH1 and PMS2 | 76 (61.8%) | 33 (73.3%) | 43 (55.1%) |
| Loss of MSH2 and MSH6 | 23 (18.7%) | 9 (20.0%) | 14 (17.9%) |
| Loss of MSH6 | 24 (19.5%) | 3 (6.7%) | 21 (26.9%) |
| **Adjuvant therapy** |  |  |  |
| Any adjuvant therapy  ( platinum-based  chemotherapy,  radiotherapy) | 62 (50.4%) | 20 (44.4%) | 42 (53.8%) |
| No further treatment | 61 (49.6%) | 25 (55.5%) | 36 (46.2%) |

Supplementary table 2. Clinicopathological characteristics of MMRd endometrial cancers from study cohort 1 and study cohort 2. MMRd, MMR deficiency.
