# Supplementary material for: High-plex spatial transcriptomic profiling reveals distinct immune components and the HLA class I/DNMT3A/CD8 modulatory axis in mismatch repair-deficient endometrial cancer
Source: Cell Oncol (Dordr). 2023 Oct 17;47(2):573–85. doi: 10.1007/s13402-023-00885-8 (PMC11090934; doi:10.1007/s13402-023-00885-8)
Supplement: Supplementary file 19 — (DOCX 21 kb) [file 13402_2023_885_MOESM19_ESM.docx]

| **Sample** | **MMR status** | **CD8+TILs/HPF** | **CD8+TILs infiltration status** |
| --- | --- | --- | --- |
| ET20-2 | MMRd | 50 | hot |
| ET20-6 | MMRd | 17 | cold |
| ET20-13 | MMRd | 196 | hot |
| ET20-37 | MMRd | 18 | cold |
| ET20-40 | MMRd | 126 | hot |
| ET20-84 | MMRd | 25 | hot |
| ET20-85 | MMRd | 61 | hot |
| ET20-88 | MMRd | 22 | hot |
| ET20-98 | MMRd | 110 | hot |
| ET19-2 | MMRd | 83 | hot |
| ET19-3 | MMRd | 50 | hot |
| ET19-4 | MMRd | 11 | cold |
| ET19-7 | MMRd | 23 | hot |
| ET19-9 | MMRd | 118 | hot |
| ET19-11 | MMRd | 22 | hot |
| ET19-15 | MMRd | 15 | cold |
| ET19-23 | MMRd | 13 | cold |
| ET19-31 | MMRd | 42 | hot |
| ET19-33 | MMRd | 130 | hot |
| ET19-34 | MMRd | 22 | hot |
| ET19-35 | MMRd | 11 | cold |
| ET19-44 | MMRd | 29 | hot |
| ET19-53 | MMRd | 49 | hot |
| ET19-54 | MMRd | 16 | cold |
| ET19-55 | MMRd | 70 | hot |
| ET19-65 | MMRd | 103 | hot |
| ET19-67 | MMRd | 25 | hot |
| ET19-69 | MMRd | 11 | cold |
| ET19-76 | MMRd | 67 | hot |
| ET19-86 | MMRd | 33 | hot |
| ET19-88 | MMRd | 39 | hot |
| ET19-90 | MMRd | 17 | cold |
| ET19-94 | MMRd | 34 | hot |
| ET19-102 | MMRd | 12 | cold |
| ET19-105 | MMRd | 26 | hot |
| ET19-106 | MMRd | 3 | cold |
| ET19-108 | MMRd | 18 | cold |
| ET19-109 | MMRd | 23 | hot |
| ET19-115 | MMRd | 14 | cold |
| ET20-102 | MMRd | 112 | hot |
| ET21-01 | MMRd | 106 | hot |
| ET21-06 | MMRd | 31 | hot |
| ET21-12 | MMRd | 3 | cold |
| ET21-14 | MMRd | 81 | hot |
| ET21-23 | MMRd | 29 | hot |
| ET20-25 | MMRp | 21 | hot |
| ET20-58 | MMRp | 2 | cold |
| ET20-8 | MMRp | 154 | hot |
| ET20-10 | MMRp | 6 | cold |
| ET20-11 | MMRp | 17 | cold |
| ET20-12 | MMRp | 11 | cold |
| ET20-14 | MMRp | 3 | cold |
| ET20-15 | MMRp | 5 | cold |
| ET20-27 | MMRp | 10 | cold |
| ET20-28 | MMRp | 13 | cold |
| ET20-82 | MMRp | 6 | cold |
| ET20-30 | MMRp | 31 | hot |
| ET20-34 | MMRp | 21 | hot |
| ET20-35 | MMRp | 15 | cold |
| ET20-36 | MMRp | 127 | hot |
| ET20-39 | MMRp | 16 | cold |
| ET20-44 | MMRp | 4 | cold |
| ET20-46 | MMRp | 24 | hot |
| ET20-65 | MMRp | 11 | cold |
| ET20-49 | MMRp | 33 | hot |
| ET19-87 | MMRp | 15 | cold |
| ET19-38 | MMRp | 7 | cold |
| ET20-71 | MMRp | 3 | cold |
| ET20-75 | MMRp | 29 | hot |
| ET20-77 | MMRp | 46 | hot |
| ET20-91 | MMRp | 1 | cold |
| ET19-8 | MMRp | 21 | hot |
| ET19-12 | MMRp | 5 | cold |
| ET19-13 | MMRp | 7 | cold |
| ET19-18 | MMRp | 12 | cold |
| ET19-25 | MMRp | 59 | hot |
| ET19-37 | MMRp | 6 | cold |
| ET19-43 | MMRp | 18 | cold |
| ET19-47 | MMRp | 21 | hot |
| ET19-50 | MMRp | 11 | cold |
| ET19-61 | MMRp | 0 | cold |
| ET19-110 | MMRp | 21 | hot |
| ET20-110 | MMRp | 4 | cold |
| ET21-03 | MMRp | 10 | cold |
| ET21-07 | MMRp | 69 | hot |
