# Supplementary material for: High-plex spatial transcriptomic profiling reveals distinct immune components and the HLA class I/DNMT3A/CD8 modulatory axis in mismatch repair-deficient endometrial cancer
Source: Cell Oncol (Dordr). 2023 Oct 17;47(2):573–85. doi: 10.1007/s13402-023-00885-8 (PMC11090934; doi:10.1007/s13402-023-00885-8)
Supplement: Supplementary file 21 — (DOCX 15 kb) [file 13402_2023_885_MOESM21_ESM.docx]

Supplementary table 5. Top 10 signaling pathways and participated genes from the 14-core gene panel identified by DSP.

| **Signaling pathway** | **Gene** |
| --- | --- |
| Type II Interferon Signaling | HLA-B/IRF9 |
| Phagocytosis | HLA-B/HLA-DMA/WIPF1/RAC2 |
| MHC Class I Antigen Presentation | HLA-B/TAPBP |
| Type I Interferon Signaling | HLA-B/IRF9 |
| MHC Class II Antigen Presentation | HLA-DMA |
| Lymphocyte Trafficking | HLA-B/HLA-DMA/CD4/RAC2 |
| NLR Signaling | IRF9/IFI16 |
| Lymphocyte Regulation | HLA-B |
| Neutrophil Degranulation | HLA-B/FCER1G/CD53 |
| TCR Signaling | CD4 |
